# Neutrophil Counts and Initial Presentation of 12 Cardiovascular Diseases: A CALIBER Cohort Study

**DOI:** 10.1016/j.jacc.2016.12.022

**Published:** 2017-03-07

**Authors:** Anoop Dinesh Shah, Spiros Denaxas, Owen Nicholas, Aroon D. Hingorani, Harry Hemingway

**Affiliations:** a Farr Institute of Health Informatics Research, UCL Institute of Health Informatics, University College London, London, United Kingdom; b University College London Hospitals NHS Foundation Trust, London, United Kingdom; c National Institute for Cardiovascular Outcomes Research, UCL Institute of Cardiovascular Science, University College London, London, United Kingdom

**Keywords:** disease mechanisms, electronic health records, epidemiology, incidence, inflammation, CI, confidence interval, CVD, cardiovascular disease, HF, heart failure, HR, hazard ratio, IQR, interquartile range, MI, myocardial infarction, PAD, peripheral arterial disease

## Abstract

**Background:**

Neutrophil counts are a ubiquitous measure of inflammation, but previous studies on their association with cardiovascular disease (CVD) were limited by small numbers of patients or a narrow range of endpoints.

**Objectives:**

This study investigated associations of clinically recorded neutrophil counts with initial presentation for a range of CVDs.

**Methods:**

We used linked primary care, hospitalization, disease registry, and mortality data in England. We included people 30 years or older with complete blood counts performed in usual clinical care and no history of CVD. We used Cox models to estimate cause-specific hazard ratios (HRs) for 12 CVDs, adjusted for cardiovascular risk factors and acute conditions affecting neutrophil counts (such as infections and cancer).

**Results:**

Among 775,231 individuals in the cohort, 154,179 had complete blood counts performed under acute conditions and 621,052 when they were stable. Over a median 3.8 years of follow-up, 55,004 individuals developed CVD. Adjusted HRs comparing neutrophil counts 6 to 7 versus 2 to 3 × 10^9^/l (both within the ‘normal’ range) showed strong associations with heart failure (HR: 2.04; 95% confidence interval [CI]: 1.82 to 2.29), peripheral arterial disease (HR: 1.95; 95% CI: 1.72 to 2.21), unheralded coronary death (HR: 1.78; 95% CI: 1.51 to 2.10), abdominal aortic aneurysm (HR: 1.72; 95% CI: 1.34 to 2.21), and nonfatal myocardial infarction (HR: 1.58; 95% CI: 1.42 to 1.76). These associations were linear, with greater risk even among individuals with neutrophil counts of 3 to 4 versus 2 to 3 × 10^9^/l. There was a weak association with ischemic stroke (HR: 1.36; 95% CI: 1.17 to 1.57), but no association with stable angina or intracerebral hemorrhage.

**Conclusions:**

Neutrophil counts were strongly associated with the incidence of some CVDs, but not others, even within the normal range, consistent with underlying disease mechanisms differing across CVDs. (White Blood Cell Counts and Onset of Cardiovascular Diseases: a CALIBER Study [CALIBER]; NCT02014610)

The most numerous type of white blood cell, neutrophils, play a major role in inflammation. Neutrophil count is used routinely as a biomarker of acute infection and inflammation, but not in cardiology. Chronic inflammation contributes to atherosclerosis and cardiovascular diseases (CVD) 1, 2 but, compared with other inflammatory biomarkers such as C-reactive protein (3) and interleukin-6 (4), neutrophil counts have been little studied in relation to long-term CVD risk, even though they are available at scale in clinically collected electronic health record data.

Previous studies have shown that high neutrophil counts are associated with an higher incidence of coronary disease (5), heart failure (HF) (6), and stroke (7) (Online Table 1). However, these studies were too small to examine thresholds or shape of the association. No study used a clinically recorded measure of neutrophil count, which is important to understand the relevance of findings to usual practice, or studied associations with peripheral vascular diseases.

This study investigated the association of neutrophil counts with initial presentation of 12 CVDs in a large, population-based cohort from a linked electronic health record database: the CALIBER program (Clinical Research Using Linked Bespoke Studies and Electronic Health Records) (8). CALIBER has been extensively validated, replicating known prospective associations of CVDs with sex (9), smoking (10), blood pressure (11), socioeconomic deprivation (12), and type 2 diabetes (13).

## Methods

We used the same study cohort as our study on the association of eosinophil and lymphocyte counts with incidence of CVD (14). The study population was drawn from the CALIBER program (8), which links 4 sources of electronic health data in England: primary care health records (coded diagnoses, clinical measurements, and prescriptions) from 244 general practices contributing to the Clinical Practice Research Datalink; coded hospital discharges (Hospital Episode Statistics); the Myocardial Ischemia National Audit Project (MINAP); and death registrations (Online Appendix). CALIBER includes about 4% of the population of England (15) and is representative in terms of age, sex, ethnicity, and mortality (8).

The study period was January 1998 to March 2010, and individuals were eligible for inclusion when they were at least 30 years of age and had been registered for at least 1 year in a practice that met research data recording standards. The study start date (index date) for each participant was the date of the first complete blood count recorded in the primary care record while the participant was eligible. Persons with a prior history of CVD and women with a pregnancy record within 6 months of the start of the study were excluded.

Approval was granted by the Independent Scientific Advisory Committee of the Medicines and Healthcare Products Regulatory Agency (protocol 12_153R) and the MINAP Academic Group.

The main exposure was the neutrophil count (part of the complete blood count) as recorded in primary care. We investigated the neutrophil count initially as a categorical variable to avoid assuming a particular shape for association with CVD. We wished to specifically look at associations with ‘normal’ as well as extreme neutrophil counts; there is no consensus definition for the normal range, but many laboratories quote the range of 2 to 7 × 10^9^/l (16). This lent itself to convenient 5-level categorization within the ‘normal’ range. All category intervals were closed at the lower bound and open at the upper bound (i.e., ‘2 to 3’ includes 2 but not 3).

White cell counts can be affected by infections, autoimmune diseases, medication, and hematologic conditions. We classified the patient state at the time of the blood test as acute or stable. An acute clinical state was defined as any of the following conditions: in hospital on the date of blood test; vaccination in the previous 7 days; anemia diagnosis within the previous 30 days; symptoms or diagnosis of infection within the previous 30 days; prior diagnosis of myelodysplastic syndrome; hemoglobinopathy, cancer chemotherapy, or injection of granulocyte colony-stimulating factor within the previous 6 months; or the use of drugs affecting the immune system, such as methotrexate or steroids, within the previous 3 months. Patients were considered stable if they did not fulfill the criteria for an acute clinical state. Patients on dialysis, those with human immunodeficiency virus infection, or a history of splenectomy were excluded from this study, because their neutrophil counts may be difficult to interpret. These criteria were based on those proposed by the eMERGE (Electronic Medical Records and Genomics) consortium for studying the genetic determinants of white cell counts (17) (further details in the Online Appendix).

In secondary analyses, we explored associations between onset of CVD and the mean of the first 2 stable measurements of neutrophil count taken since the start of eligibility. We extracted demographic variables, cardiovascular risk factors, comorbidities, and acute conditions and prescriptions around the time of the blood test from the primary care record. For continuous covariates, we used the most recent value in the year before or up to 1 day after the complete blood count measurement. We also extracted the first measurement after this time window and the last measurement before the time window, along with the timing of these measurements relative to the index date, to use as auxiliary variables for multiple imputation. We also used comorbidity information from hospitalization records.

Individuals were followed until initial presentation of CVD, death, or transfer out of the practice. The primary endpoint was the first record of 1 of the following 12 initial cardiovascular presentations in any of the data sources: coronary artery disease (stable angina, unstable angina, nonfatal myocardial infarction [MI], unheralded coronary death), HF, transient ischemic attack, ischemic or hemorrhagic stroke, subarachnoid hemorrhage, peripheral arterial disease (PAD), abdominal aortic aneurysm, or a composite of ventricular arrhythmia, implantable cardioverter-defibrillator, cardiac arrest, or sudden cardiac death. Nonspecific coronary artery disease and nonspecific stroke were also included in the analysis as 2 additional endpoints, although they are artefacts of imprecise coding rather than separate disease entities. Any events occurring after the first cardiovascular presentation were ignored. Endpoint definitions are described in the Online Appendix and phenotyping algorithms are available on the CALIBER web portal (website in the Online Appendix). We analyzed all-cause mortality and a composite of all initial cardiovascular presentations as secondary endpoints.

### Statistical analysis

We examined associations of CVD with neutrophil counts initially as a categorical variable. If the shape of the association was found to be linear, we also performed analyses with neutrophil count as a continuous variable. We generated cumulative incidence curves by category of neutrophil count under a competing risks framework. We used Cox proportional hazards models to estimate cause-specific hazards for each of the 14 cardiovascular endpoints. Hazard ratios (HRs) were adjusted for age (linear and quadratic), sex, age–sex interaction, index of multiple deprivation, ethnicity, smoking status, diabetes, body mass index, systolic blood pressure, estimated glomerular filtration rate, high-density lipoprotein cholesterol, total cholesterol, atrial fibrillation, inflammatory conditions (autoimmune conditions, chronic obstructive pulmonary disease, or inflammatory bowel disease), cancer, statin use, blood pressure medication, and acute conditions at the time of blood testing. The baseline hazard was stratified by practice and sex.

We plotted Schoenfeld residuals to assess the proportional hazards assumption, and split the follow-up time if HRs changed over time. We examined for interactions with age, sex, smoking, and acute clinical state. We handled missing baseline covariate data by multiple imputation using random forest (18), as described in the Online Appendix. We explored additional adjustments for eosinophil and lymphocyte counts, which are also associated with incidence of CVD (14) (each was included as a 5-category variable). We used a Bonferroni correction for 14 comparisons to designate the level of statistical significance as 0.0036, and expressed p values in categories: <0.05 (suggestive of a trend), <0.0036 (statistically significant), and <0.0001 (strong evidence).

## Results

We included 621,052 patients with neutrophil counts while clinically stable and 154,179 patients with neutrophil counts performed during acute illness or treatment (Online Figure 1, Online Table 2). We observed 55,004 initial presentations of CVD over a median follow-up of 3.8 years (interquartile range [IQR]: 1.7 to 6.0 years).

**Figure 1:**
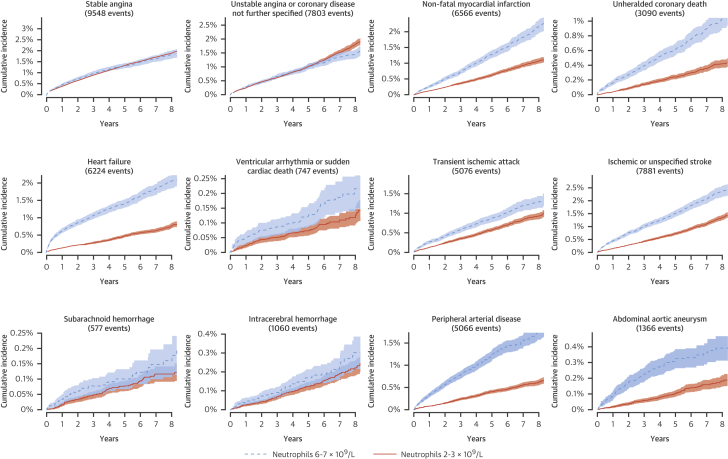
Cumulative Incidence Curves For cardiovascular presentations among people without prior cardiovascular disease (CVD), crude cumulative incidence curves are shown for the highest and lowest categories of neutrophil count within the normal range. An artefact of imprecise coding rather than a clinical diagnosis, ‘nonspecific coronary disease’ was combined with unstable angina. Similarly, nonspecific stroke was combined with ischemic stroke. The plots show that, for myocardial infarction, heart failure, ischemic stroke, peripheral arterial disease (PAD), and abdominal aortic aneurysm, the incidence was greater among people with higher neutrophil counts.

Patients with higher neutrophil count were more likely to smoke, live in a socioeconomically deprived area, and have comorbidities such as diabetes, asthma, chronic obstructive pulmonary disease, connective tissue diseases, and inflammatory bowel disease (Table 1). Patients with neutrophil counts above or below the limits of normal were more likely to have an acute state at the time of blood testing than those with neutrophil counts within the normal range (27.5% [22,678 of 82,376] vs. 19.0% [131,501 of 692,855]; p < 0.0001). Symptoms or diagnosis of infection were the most frequent reason for the patient’s condition to be classified as acute (Online Table 3). People with neutrophil counts of 6 to 7 × 10^9^/l (at the upper end of the normal range) had a higher incidence of nonfatal MI, unheralded coronary death, HF, PAD, and abdominal aortic aneurysm compared with people with neutrophil counts of 2 to 3 × 10^9^/l (Figure 1). The risk difference appeared to be greatest for the first few months (Online Table 4). Individuals with higher neutrophil counts were relatively more likely to present with unheralded coronary death, HF, or PAD than stable angina (Online Table 5).

**Figure 2:**
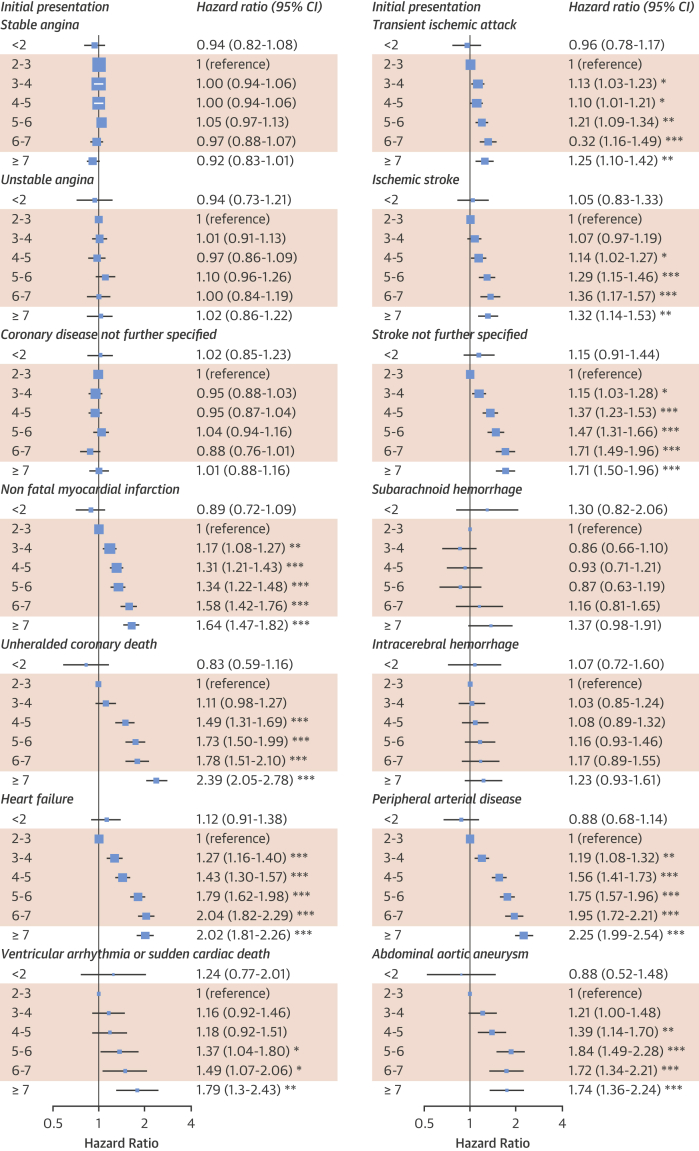
Association of Neutrophil Count With Initial CVD Presentation Neutrophil count categories influenced cause-specific adjusted hazard ratios for cardiovascular presentations among people without prior cardiovascular disease (CVD). Hazard ratios were adjusted for age, sex, deprivation, ethnicity, smoking, diabetes, systolic blood pressure (SBP), blood pressure medication, body mass index (BMI), total cholesterol, high-density lipoprotein cholesterol (HDL-C), statin use, estimated glomerular filtration rate (eGFR), atrial fibrillation (AF), autoimmune conditions, inflammatory bowel disease (IBD), chronic obstructive pulmonary disease (COPD), cancer, and acute conditions at the time of blood testing. Shaded = normal range. *p < 0.05; **p < 0.0036; ***p < 0.0001. CI = confidence interval; other abbreviations as in Figure 1.

There were strong, specific associations between neutrophil counts and different initial presentations of CVD (Figure 2). Adjusted HRs comparing neutrophil counts 6 to 7 versus 2 to 3 × 10^9^/l showed strong associations with HF (HR: 2.04; 95% confidence interval [CI]: 1.82 to 2.29), unheralded coronary death (HR: 1.78; 95% CI: 1.51 to 2.10), and nonfatal MI (HR: 1.58; 95% CI: 1.42 to 1.76), but not stable angina (HR: 0.97; 95% CI: 0.88 to 1.07) or unstable angina (HR: 1.00; 95% CI: 0.84 to 1.19) (Figure 2). The association with ischemic stroke was weak (HR: 1.36; 95% CI: 1.17 to 1.57) and there was no association with hemorrhagic stroke. There were strong associations with PAD (HR: 1.95; 95% CI: 1.72 to 2.21) and abdominal aortic aneurysm (HR: 1.72; 95% CI: 1.34 to 2.21) (Figure 2). The associations were stronger in models adjusted only for age and sex (Online Figure 2).

There was a strong association of neutrophil count with noncardiovascular death when comparing neutrophil counts of 6 to 7 versus 2 to 3 × 10^9^/l (HR: 2.01; 95% CI: 1.91 to 2.11), with a higher proportion of deaths due to pneumonia or chronic obstructive pulmonary disease (Online Table 4). There was also an association of higher neutrophil count with the composite of CVD (Online Figure 3) and all-cause mortality (Online Figure 4). Neutrophil counts of <2 × 10^9^/l were associated with greater risk of noncardiovascular death (compared with 2 to 3 × 10^9^/l: HR: 1.52; 95% CI: 1.41 to 1.63), but were not associated with greater risk of any presentation of CVD (Online Figure 3).

Because the associations between neutrophil counts and cardiovascular presentations were monotonic and linear, we treated neutrophil count as a linear variable in subsequent modeling. We found stronger associations within the normal range (Online Figure 5), but no interaction with smoking status (Online Figure 6). Additional adjustment for eosinophil and lymphocyte counts did not alter the estimates (Online Figure 7).

**Figure 3:**
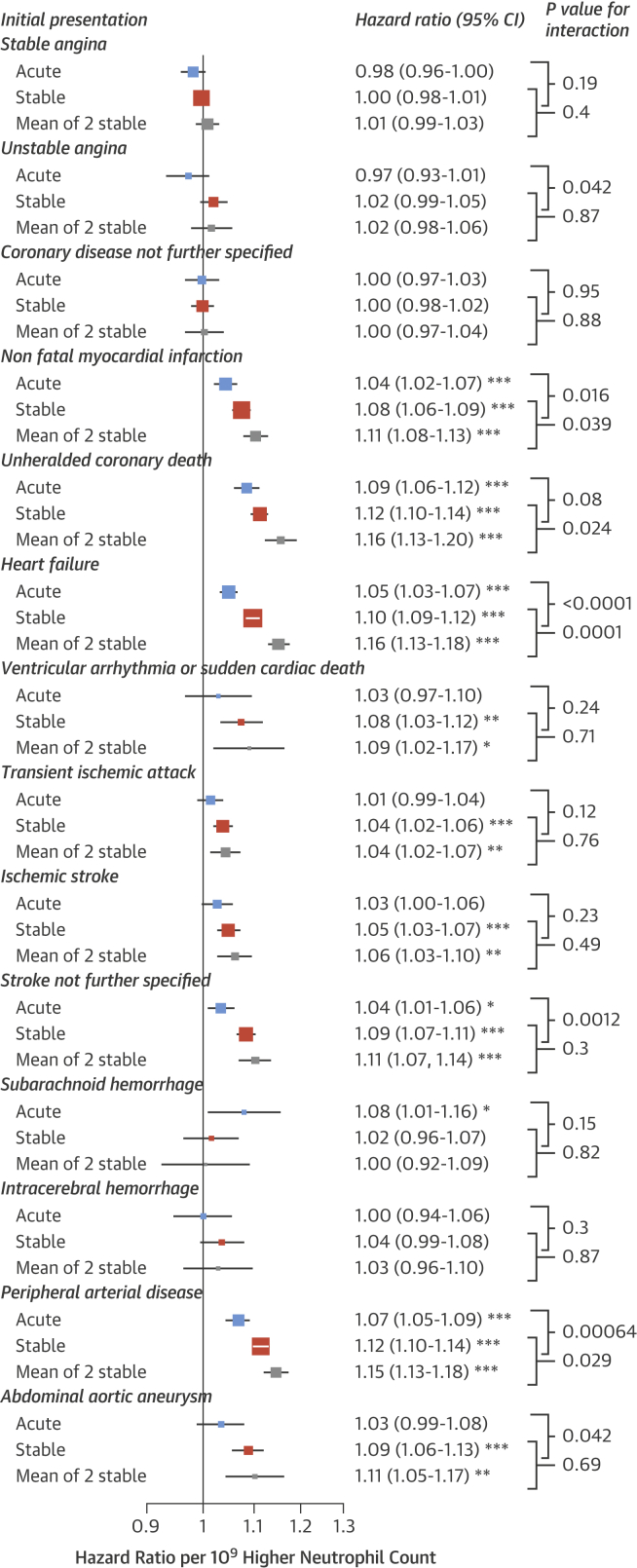
CVD and Neutrophil Counts by Clinical State at Blood Sampling Hazard ratios for initial presentation of CVDs by neutrophil count varied by clinical state at the time of blood sampling. ‘Mean of 2 stable’ refers to the mean of 2 consecutive neutrophil counts performed in a stable clinical state. Hazard ratios are adjusted for age, sex, socioeconomic deprivation, ethnicity, smoking, diabetes, SBP, blood pressure medication, BMI, total cholesterol, HDL-C, statin use, eGFR, AF, autoimmune conditions, IBD, COPD, and cancer. *p < 0.05; **p < 0.0036; ***p < 0.0001. Abbreviations as in Figures 1 and 2.

There was some evidence that associations between neutrophil counts and cardiovascular presentations were stronger for stable compared with acute measurements, particularly for PAD and HF (Figure 3). Associations were further strengthened when we used the mean of 2 consecutive stable neutrophil counts, which were available in 393,543 patients and were taken at a median of 1.4 years apart (IQR: 0.6 to 2.7 years) (Figure 3). There was considerable variability but minimal trend over time between repeat measurements of stable neutrophil counts; the SD of differences between 2 consecutive measurements was 1.67 × 10^9^/l, the correlation coefficient was 0.568, and the mean rate of change was a decrease of 0.014 per year (95% CI: 0.011 to 0.017) (Online Figure 8).

We found that hazards were nonproportional for some of the endpoints. We therefore split the follow-up time by 6 months, and found that neutrophil counts were more strongly associated with HF, unheralded coronary death, and ischemic stroke in the first 6 months (Online Figure 9). Associations with coronary endpoints, HF, and PAD were stronger among younger patients (Online Figure 10). The association between neutrophil count and initial presentation with HF was stronger in men than women (HR per 10^9^/l higher neutrophil count: 1.10 vs. 1.07; p = 0.001). There was an association between neutrophil count and initial presentation with transient ischemic attack in women but not men (HR: 1.05 vs. 1.00; p = 0.007) (Online Figure 11).

## Discussion

Neutrophil counts within the range clinicians currently consider normal had strong linear associations with some, but not all, CVDs in a population-based cohort. We found a greater cumulative incidence of unheralded coronary death, nonfatal MI, HF, PAD, and abdominal aortic aneurysm in patients with neutrophil counts at the upper end of the normal range (Figure 1), and these associations were confirmed in multivariable survival models. Our findings were consistent with those of previous, smaller studies that showed a positive association of higher neutrophil count with HF (6) and a moderate association with cerebral infarction (7); a key novel finding of our study is the association with PAD and abdominal aortic aneurysm, which has not been reported previously. In contrast with MI, we found that neutrophil count was not associated with a greater incidence of stable or unstable angina. This study’s large sample size (>700,000 patients) made it possible to investigate less common CVDs; we showed lack of association with intracerebral hemorrhage and subarachnoid hemorrhage.

### Specificity and strength of associations

We disaggregated CVD into pathologically diverse initial presentations to help elucidate the mechanistic role of neutrophils. The stronger association seen with MI than angina suggests that as well being involved in inflammation and atherosclerosis, neutrophils also increase the risk of arterial thrombosis (Central Illustration). Possible mechanisms include interactions with the endothelium and platelets, and overactivity of neutrophil extracellular traps (19). We also observed associations of higher neutrophil count with noncardiovascular and overall mortality, suggesting that chronic inflammation has noncardiovascular adverse effects that warrant further study.

We examined associations with other leukocyte subtypes and found that monocyte counts had a similar pattern of associations with initial presentations of CVD to neutrophils, but the associations were not as strong (Online Figure 12). Eosinophil and lymphocyte counts have a different pattern of association with initial presentations of CVD (14), but additional adjustment for these leukocyte subtypes did not alter the results for neutrophils (Online Figure 7), suggesting that these associations are independent.

Neutrophil count has a similar strength and shape of association with CVD as systolic blood pressure, with no evidence of a threshold effect among higher neutrophil counts. A moderate chronic elevation of systolic blood pressure of 60 mm Hg (e.g., 180 mm Hg instead of 120 mm Hg) is associated with an approximate doubling of the risk of incident HF (scaled from Rapsomaniki et al. [11]), which is comparable with the HR comparing the upper and lower ends of the normal range for neutrophil count (HR: 2.04; 95% CI: 1.82 to 2.29) (Figure 2).

We also found that associations were stronger among patients with neutrophil counts taken under stable conditions, and when the mean of 2 neutrophil counts was used; this finding provides further evidence for the relevance of a patient’s chronic inflammatory state.

### Targets and interventions

Reducing chronic inflammation could be a potential therapeutic avenue in atherosclerotic disease. Our study could not ascertain whether it is circulating neutrophils per se that confer the additional risk or the underlying inflammatory state of which the neutrophil count is a marker (20). Investigation of causal mechanisms could involve epidemiological studies of upstream determinants of neutrophil counts, such as granulocyte colony-stimulating factor, interleukin-17, and interleukin-23 (21). Mendelian randomization studies using single nucleotide polymorphisms for genes associated with neutrophil count, such as those identified in the 17q21 region (22), might also help to evaluate causal relevance.

Colchicine, which has a range of actions on many cell types, including inhibiting microtubule formation in neutrophils, reduced the incidence of acute coronary syndrome and stroke in a trial among patients with stable coronary disease (23). Trials are underway to investigate whether anti-inflammatory agents such as methotrexate (24) or canakinumab (a human monoclonal antibody against interleukin-1-beta) (25) can prevent CVD events in high-risk individuals.

Smoking causes an elevation of neutrophil counts and is associated more strongly with MI than stable angina (10), like neutrophil counts. A clinical trial of smoking cessation found that it reduced neutrophil count by 1.0 × 10^9^/l (26). We adjusted for smoking in the main analysis, but if an increased neutrophil count (or the underlying chronic inflammation it represents) is on a causal pathway linking smoking to CVD, we might be overadjusting for smoking, thereby underestimating the component of cardiovascular risk conveyed by inflammation or neutrophils.

Other modifiable factors that can increase the level of chronic low-grade inflammation include air pollution (27), obesity (28), lack of exercise (29), and periodontal disease (30).

### Study limitations

Although our study had strengths—its large size, population base, and extensive adjustment for potential confounders—it also had important limitations. Our distinction between acute and stable patients was crude, but the similarity of findings in both groups was reassuring. As with any observational study, the results cannot be taken to imply causation due to the possibility of residual confounding. The measurement of neutrophil counts was undertaken in usual clinical care, and by different laboratories without study-wide protocols. Heterogeneity of measurement methods and heterogeneity among the study population itself may have led to biased estimates of association, but these would tend to be underestimates. The ascertainment of endpoints was in coded clinical data without manual endpoint adjudication. All the data sources used in this study missed some events (15); however, any errors in endpoint recording were likely to be nondifferential in relation to the neutrophil count. Because our study was based on electronic health records, some values of baseline variables were missing for some patients. However, we obtained similar results by imputing missing data using 2 different methods of multiple imputation.

### Clinical implications

Our results suggest that the current clinical practice of labeling the range of neutrophil counts 2 to 7 × 10^9^/l as normal—and ignoring any risk information conveyed by the actual value—should be reconsidered. Neutrophil counts are a measure of a patient’s chronic inflammatory state, which relates to cardiovascular risk and can be modified. Clinicians should look out for treatable causes of chronic inflammation in such patients, such as periodontal disease (30).

Further research on the associations of neutrophil counts with CVD is warranted, including investigation of diurnal and seasonal variations and utility in risk prediction (20). Other biomarkers of inflammation are associated with greater risk of coronary disease (3), and U.S. guidelines recommend that high-sensitivity C-reactive protein can be measured to inform decisions on cardiovascular risk management in uncertain cases (31). An advantage of using the neutrophil count for this purpose, over other inflammatory biomarkers, is that it is already measured and would not incur any additional costs for testing.

Third, neutrophil counts could be considered as a means of monitoring a patient’s progress or as a surrogate endpoint in trials investigating anti-inflammatory interventions to reduce cardiovascular risk.

## Conclusions

Clinically recorded neutrophil counts were strongly associated with the incidence of specific CVDs, even within the normal range. The neutrophil count should be further evaluated as an inflammatory biomarker relevant to CVD.

Perspectives **COMPETENCY IN MEDICAL KNOWLEDGE:** Peripheral blood neutrophil counts within the normal range in people without prior CVD range are associated linearly with risk of developing MI, ischemic stroke, HF, peripheral artery disease, and abdominal aortic aneurysm.

**TRANSLATIONAL OUTLOOK:** Future investigations, such as Mendelian randomization studies, should seek to understand the causal relevance of neutrophils to various forms of CVD, and ascertain whether therapies targeting neutrophils have clinical value in preventing these diseases.

## Figures and Tables

**Central Illustration:**
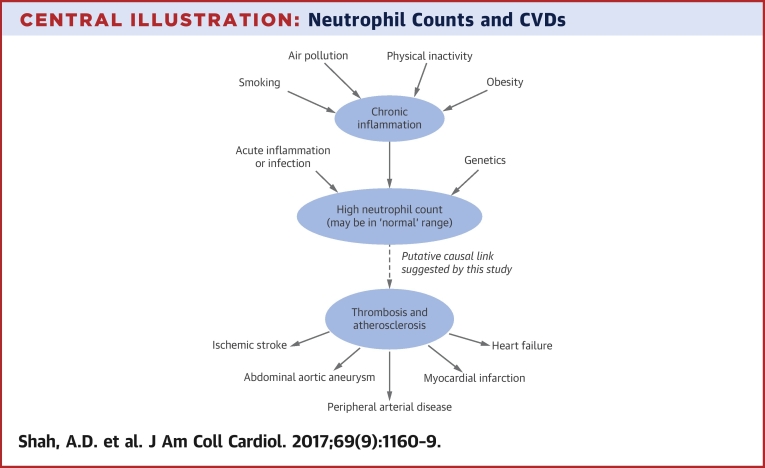
Neutrophil Counts and CVDs Potential causal pathways are depicted linking chronic inflammation, neutrophil counts, and onset of cardiovascular diseases (CVD). Environmental and behavioral risk factors such as smoking, air pollution, and physical inactivity contribute to chronic inflammation. An inflammatory state results in a higher neutrophil count, which may be causally linked with increased risk of certain cardiovascular conditions.

**Table 1:** Patient Characteristics∗

	Neutrophil Count					p Value for Trend
	Below Normal Range	Within Normal Range			Above Normal Range	
Neutrophils, × 10^9^/l	<2	2–3	3–6	6–7	≥7	
N	26,588	154,863	489,143	48,849	55,788	
Female	16,592 (62.4)	89,956 (58.1)	288,221 (58.9)	31,465 (64.4)	35,722 (64.0)	<0.0001
Age, yrs	51.3 (40.6–61.8)	53 (42.2–63.1)	52.9 (42.0–65.3)	49.8 (38.8–65)	48.2 (36.9–64.7)	<0.0001
Most deprived quintile	4,513 (17.0)	21,722 (14.1)	84,740 (17.4)	10,158 (20.9)	11,838 (21.3)	<0.0001
Acute condition at time of blood test	5,670 (21.3)	26,932 (17.4)	92,969 (19.0)	11,600 (23.7)	17,008 (30.5)	<0.0001
Ethnicity						
White	12,945 (80.3)	82,468 (90.8)	283,495 (93.5)	30,174 (94.5)	35,952 (95.4)	<0.0001
South Asian	327 (2.0)	2,482 (2.7)	9,083 (3.0)	813 (2.5)	745 (2.0)	<0.0001
Black	2,229 (13.8)	3,224 (3.6)	3,936 (1.3)	310 (1.0)	275 (0.7)	<0.0001
Other	626 (3.9)	2,624 (2.9)	6,740 (2.2)	620 (1.9)	710 (1.9)	<0.0001
Missing	10,461 (39.3)	64,065 (41.4)	185,889 (38.0)	16,932 (34.7)	18,106 (32.5)	<0.0001
Full blood count parameters on index date						
Eosinophils, × 10^9^/l	0.1 (0.09–0.2)	0.13 (0.1–0.2)	0.2 (0.1–0.27)	0.2 (0.1–0.3)	0.16 (0.1–0.27)	<0.0001
Lymphocytes, × 10^9^/l	1.7 (1.36–2.07)	1.8 (1.5–2.2)	2.0 (1.61–2.49)	2.1 (1.69–2.7)	2.05 (1.58–2.63)	<0.0001
Monocytes, × 10^9^/l	0.34 (0.29–0.42)	0.4 (0.3–0.5)	0.5 (0.4–0.6)	0.6 (0.5–0.74)	0.7 (0.5–0.9)	<0.0001
Basophils, × 10^9^/l	0.01 (0–0.03)	0.02 (0–0.05)	0.03 (0–0.08)	0.03 (0–0.1)	0.03 (0–0.1)	<0.0001
Hemoglobin, g/dl	13.5 (12.6–14.5)	13.9 (13–14.8)	14.0 (13.1–15)	13.8 (12.8–14.9)	13.7 (12.4–14.8)	<0.0001
Platelets, × 10^9^/l	225 (190–263)	241 (207–279)	262 (224–306)	286 (242–338)	298 (249–360)	<0.0001
Smoking status						
Never	16,657 (66.1)	91,447 (62.1)	240,087 (51.7)	18,758 (40.7)	19,753 (37.7)	<0.0001
Former	5,798 (23.0)	36,839 (25.0)	114,129 (24.6)	9,736 (21.1)	10,697 (20.4)	<0.0001
Current	2,754 (10.9)	18,938 (12.9)	109,796 (23.7)	17,615 (38.2)	21,918 (41.9)	<0.0001
Missing	1,379 (5.2)	7,639 (4.9)	25,131 (5.1)	2,740 (5.6)	3,420 (6.1)	<0.0001
Most recent value within 1 yr before the index date						
Systolic BP	131 (120–145)	135 (120–148)	138 (123–150)	135 (120–150)	130 (120–146)	<0.0001
BMI	25.4 (22.7–28.7)	26.3 (23.4–29.8)	27.4 (24.1–31.5)	27.1 (23.4–32.1)	26.4 (22.9–31.1)	<0.0001
Total cholesterol	5.4 (4.7–6.2)	5.5 (4.8–6.3)	5.5 (4.8–6.2)	5.3 (4.6–6.1)	5.3 (4.5–6.1)	<0.0001
HDL cholesterol	1.53 (1.24–1.9)	1.47 (1.20–1.79)	1.35 (1.10–1.62)	1.30 (1.08–1.60)	1.3 (1.07–1.59)	<0.0001
eGFR	84.4 (71.9–97.4)	82.8 (70.7–95.2)	81.4 (68.3–94.5)	82.4 (67.7–96.7)	83.1 (67.3–97.5)	<0.0001
Diagnoses on or before index date						
Atrial fibrillation	176 (0.7)	1,044 (0.7)	5,169 (1.1)	602 (1.2)	731 (1.3)	<0.0001
Cancer	2,089 (7.9)	9,343 (6.0)	29,468 (6.0)	2,863 (5.9)	3,758 (6.7)	0.2
Diabetes	860 (3.2)	5,322 (3.4)	25,081 (5.1)	2,896 (5.9)	2,968 (5.3)	<0.0001
Asthma	2,721 (10.2)	17,046 (11.0)	61,521 (12.6)	7,165 (14.7)	8,344 (15.0)	<0.0001
COPD	178 (0.7)	1,329 (0.9)	9,545 (2.0)	1,614 (3.3)	2,355 (4.2)	<0.0001
Connective tissue disease	596 (2.2)	3,057 (2.0)	13,585 (2.8)	2,007 (4.1)	2,614 (4.7)	<0.0001
IBD	207 (0.8)	1,316 (0.8)	5,377 (1.1)	717 (1.5)	1,029 (1.8)	<0.0001
Medication use in the year before index date						
Antihypertensives	5,342 (20.1)	34,000 (22.0)	130,791 (26.7)	13,255 (27.1)	13,893 (24.9)	<0.0001
Statins	1,141 (4.3)	8,120 (5.2)	31,713 (6.5)	3,010 (6.2)	2,822 (5.1)	<0.0001

Values are n (%) or median (interquartile range) unless otherwise indicated.

BMI = body mass index; BP = blood pressure; COPD = chronic obstructive pulmonary disease; eGFR = estimated glomerular filtration rate; HDL = high-density lipoprotein; IBD = inflammatory bowel disease.

∗ Completeness of recording of continuous variables at any time (as used for imputation) was 98.8% for BP, 90.6% for body mass index, 58.9% for HDL and total cholesterol, and 57.1% for eGFR. Completeness of recording of these variables within 1 year before the index date was 65.4%, 31.3%, 31.6%, and 47.0%, respectively. Diagnoses of comorbid conditions were considered completely recorded (i.e., absence of a diagnosis code was interpreted as absence of the condition).
