# Correction

**DOI:** 10.1016/j.jacc.2017.05.026

**Published:** 2017-06-27

Shah AD, Denaxas S, Nicholas O, Hingorani AD, Hemingway H

**Neutrophil Counts and Initial Presentation of 12 Cardiovascular Diseases: A CALIBER Cohort Study**

**J Am Coll Cardiol 2017;69:1160–9.**

In Figure 2, in the Transient Ischemic Attack panel, “0.32 (1.16-1.49)” should have read “1.32 (1.16-1.49).”

The corrected Figure 2 is printed below.

**Figure 2:**
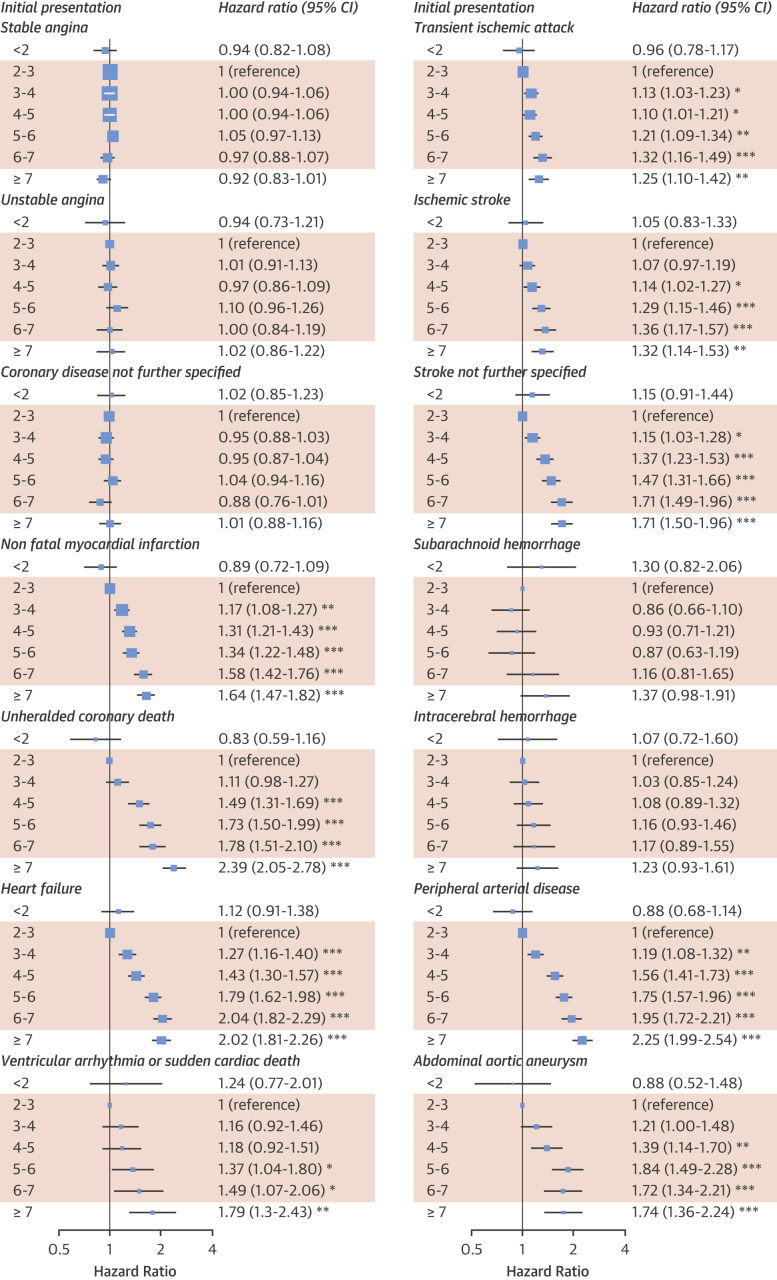
Association of Neutrophil Count With Initial CVD Presentation Neutrophil count categories influenced cause-specific adjusted hazard ratios for cardiovascular presentations among people without prior cardiovascular disease (CVD). Hazard ratios were adjusted for age, sex, deprivation, ethnicity, smoking, diabetes, systolic blood pressure (SBP), blood pressure medication, body mass index (BMI), total cholesterol, high-density lipoprotein cholesterol (HDL-C), statin use, estimated glomerular filtration rate (eGFR), atrial fibrillation (AF), autoimmune conditions, inflammatory bowel disease (IBD), chronic obstructive pulmonary disease (COPD), cancer, and acute conditions at the time of blood testing. Shaded = normal range. *p < 0.05; **p < 0.0036; ***p < 0.0001. CI = confidence interval; other abbreviations as in Figure 1.

The authors apologize for these errors.

The online version of the article has been corrected to reflect these changes.

