# Supplementary material for: Neutrophil Counts and Initial Presentation of 12 Cardiovascular Diseases: A CALIBER Cohort Study
Source: J Am Coll Cardiol. 2017 Mar 7;69(9):1160–9. doi: 10.1016/j.jacc.2016.12.022 (PMC5332591; doi:10.1016/j.jacc.2016.12.022)

Neutrophil counts and initial presentation of 12 cardiovascular diseases: a CALIBER cohort study

Online Appendix

Table of Contents

Supplementary Methods....2
- Study data sources....2
- Classification of patient state as 'acute' or 'stable' on date of blood test....2
- Endpoint definitions....2
- Survival analysis and competing risks....3
- Multiple imputation....4

References....5

Online Tables....7
- Online Table 1: Cohort studies with over 2000 participants investigating neutrophil counts and incidence of cardiovascular diseases....7
- Online Table 2: Characteristics of patients and prevalence of conditions defining 'acute' and 'stable' complete blood counts....8
- Online Table 3: Prevalence of acute conditions by category of neutrophil count....9
- Online Table 4: Endpoints by category of neutrophil count....10
- Online Table 5: Cumulative incidence of initial cardiovascular presentations....11

Online Figures....12
- Online Figure 1: Patient flow diagram....12
- Online Figure 2: Age and sex adjusted hazard ratios for the association of neutrophil counts with different initial presentations of cardiovascular diseases....13
- Online Figure 3: Association of neutrophil counts with composite cardiovascular disease and non-cardiovascular death....14
- Online Figure 4: Association of neutrophil counts with all-cause mortality....15
- Online Figure 5: Linear association of neutrophil counts with different initial presentations of cardiovascular disease…16
- Online Figure 6: Linear association of neutrophil counts with different initial presentations of cardiovascular disease, by smoking status....17
- Online Figure 7: Linear association of neutrophil counts with different initial presentations of cardiovascular disease, with and without adjustment for eosinophil and lymphocyte counts....18
- Online Figure 8: Binned scatterplot showing difference between two consecutive neutrophil counts taken when a patient was clinically 'stable'....19
- Online Figure 9: Linear association of neutrophil counts with different initial presentations of cardiovascular disease, by time since measurement....20
- Online Figure 10: Linear association of neutrophil counts with different initial presentations of cardiovascular disease, by age group....21
- Online Figure 11: Linear association of neutrophil counts with different initial presentations of cardiovascular disease, by sex....22
- Online Figure 12: Association of quintiles of monocyte counts with different initial presentations of cardiovascular disease....23

Supplementary Methods

We used the same study cohort as our recent study on the association of eosinophil and lymphocyte counts with incidence of cardiovascular diseases (1).

Study data sources

The CALIBER research platform (Cardiovascular disease research using Linked Bespoke studies and Electronic Records) (2) contains linked electronic health records from four data sources in England:

1. Primary care data from 225 general practices in the Clinical Practice Research Datalink (CPRD) (3) CPRD provides primary care data on demographics, ethnicity, health behaviours, diagnoses, investigations, procedures and prescriptions. Diagnoses are coded using the Read Clinical Terminology. (Read Terms are a major component of the SNOMED-CT terminology).
2. Hospital Episodes Statistics (HES), containing details of hospital admissions (http://www.hscic.gov.uk/hes). Diagnoses are coded using the International Statistical Classification of Diseases and Health Related Problems, 10th revision (ICD-10); interventions are coded using the Office of the Population Censuses and Surveys Classification of Interventions and Procedures (OPCS). Ethnicity as recorded during hospital attendances is also included in HES.
3. Details of acute coronary syndromes from the Myocardial Ischemia National Audit Project registry (MINAP) (4).
4. Date and ICD-10 coded cause of death from the Office for National Statistics (ONS) death registry. The index of multiple deprivation according to the patient's area of residence was also obtained from ONS.

The linkage was carried out in October 2010 by a trusted third party, using a deterministic match between the NHS number (the National Health Service unique patient identifier), date of birth, and sex. Overall, 96% of patients with a valid NHS number were successfully matched (5).

Classification of patient state as 'acute' or 'stable' on date of blood test

We used information in the primary care and hospitalization records to classify the patient state on the date of the differential leukocyte count. These criteria were based on the recommendations of the eMERGE consortium (http://phenotype.mc.vanderbilt.edu/group/emerge-phenotype-wg) for studies to identify genetic determinants of the underlying stable leukocyte count. The eMERGE criteria were modified to be suitable for a cohort study, avoiding the use of clinical events after the index date in order to avoid immortal time bias. The criteria for an 'acute' patient state were: in hospital on the date of blood test, vaccination in the previous 7 days, anemia diagnosis within the previous 30 days, symptoms or diagnosis of infection within the previous 30 days, prior diagnosis of myelodysplastic syndrome, prior diagnosis of hemoglobinopathy, cancer chemotherapy or G-CSF within 6 months before index date, or the use of drugs affecting the immune system such as methotrexate or steroids within the previous 3 months.

Endpoint definitions

Cardiovascular phenotype definitions based on the CALIBER data sources are curated on the CALIBER data portal (https://caliberresearch.org/portal). The endpoint was the first occurrence of one of the following cardiovascular presentations. If more than one endpoint was recorded on the same date, the lowest numbered endpoint was allocated.

1. **Ventricular arrhythmia or sudden cardiac death** was a composite of ventricular arrhythmias, implantable cardioverter defibrillator, and sudden cardiac death. It was defined using diagnoses and procedure codes in primary care, secondary care and death certificates.

2. **Heart failure** was defined by coded diagnoses in primary care, secondary care and death certificates.

3. **Unheralded coronary death** was death with the primary cause certified as coronary heart disease, and no

prior history of cardiovascular disease. Patients with myocardial infarction who died on the day of their infarct were considered to have unheralded coronary death.

4. **Non-fatal myocardial infarction** was defined as a disease registry diagnosis of an acute coronary syndrome with elevated troponin, or a primary or secondary care diagnosis of myocardial infarction.

5. **Unstable angina** was defined as a primary or secondary care diagnosis of unstable angina, or an acute coronary syndrome without myocardial infarction recorded in the disease registry.

6. **Stable angina** was defined by a coded diagnosis in primary or secondary care of ischemic chest pain or stable angina, a positive myocardial ischemia test, two or more prescriptions of antianginal medication, or coronary revascularization.

7. **Coronary disease not further specified** is a non-specific diagnosis of ischemic or coronary heart disease in primary or secondary care that does not fall into one of the more specific categories. This is an artefact of imprecise coding rather than a clinical diagnosis, so for cumulative incidence calculations it was combined with unstable angina.

8. **Abdominal aortic aneurysm** was defined by coded diagnoses and procedures in primary care, secondary care and death certificates.

9. **Peripheral arterial disease** includes intermittent claudication, limb ischemia or gangrene due to atherosclerotic disease in the arteries of the legs. It was defined by coded diagnoses and procedures in primary care, secondary care and death certificates.

10. **Subarachnoid hemorrhage** was defined by coded diagnoses in primary care, secondary care and death certificates.

11. **Intracerebral hemorrhage** was defined by coded diagnoses in primary care, secondary care and death certificates.

12. **Ischemic stroke** was defined using coded diagnoses in primary care, secondary care and death certificates. Patients with a procedure code for carotid endarterectomy within 90 days of a stroke of unspecified type were considered to have ischemic stroke.

13. **Stroke not further specified** is a diagnosis of stroke which does not state it is ischemic or hemorrhagic. This is an artefact of imprecise coding rather than a clinical diagnosis, so for cumulative incidence calculations it was combined with unstable angina.

14. **Transient ischemic attack** was defined by coded diagnoses in primary or secondary care.

Survival analysis and competing risks

We carried out survival analysis to model the first occurrence of any cardiovascular disease. A patient's follow-up ended when they experienced one of the cardiovascular endpoints or when they were censored. Subsequent events (e.g. myocardial infarction occurring after stable angina) were not analyzed.

The main exposure was the neutrophil count (part of the complete blood count) as recorded in primary care. If a patient had more than one measurement on a given day, the mean was taken. We investigated neutrophil counts initially as a categorical variable (<2, 2 to 3, 3 to 4, 4 to 5, 5 to 6, 6 to 7, $\geq 7 \times 10^9$/L) to avoid assuming a particular shape for the association with cardiovascular diseases. All category intervals were closed at the lower bound and open at the upper bound, i.e. '2 to 3' includes 2 but not 3. We designated the category that was most commonly lowest risk as the reference category to make the results easy to interpret; this turned out to be 2 to 3 $\times 10^9$/L. If the shape of the association was found to be linear, we also performed analyses with neutrophil count as a continuous variable.

To describe the incidence of each initial presentation over time we constructed cumulative incidence curves, taking into account the other possible initial presentations as competing events. Normal-based confidence

intervals were constructed based on Greenwood's variance formula, as implemented in the R prodlim package (http://CRAN.R-project.org/package=prodlim).

For multivariable modelling we considered using Cox models (for modelling cause-specific hazards) or the Fine and Gray model (to compare cumulative incidence curves by modelling subdistribution hazards). As the aim of this study was observational epidemiology – to explore associations rather than predict risk – we considered cause specific hazard ratios to be appropriate quantities to estimate. They should be interpreted together with cumulative incidence curves but cannot be used to predict cumulative incidence. We also found the Fine and Gray model to be more computationally intensive, and it would require significant software engineering or computing time to apply it to the large dataset used in these analyses. We used follow-up time as the timescale for the Cox models in order to investigate how the strength of association between a leukocyte count measurement and outcome varies depending on time since measurement, and determine whether it is more useful for short / medium or long term prediction.

Neutrophils are short-lived cells and are affected by acute illnesses; we found there was significant variation between repeat tests on the same individual, giving a within-person correlation of neutrophil counts taken under 'stable' conditions of 0.568. Hence inter-test variability could affect the usefulness of the neutrophil count as a cardiovascular risk marker. We did not adjust for this regression dilution bias in these analyses, because although the adjusted results would convey more accurately the strength of the biological association with the unobservable average neutrophil count, they would give a misleading impression of the predictive value of the small number of neutrophil counts available in practice for an individual patient. Instead we investigated associations with the mean of 2 neutrophil counts taken under 'stable' conditions, which would give a more precise estimate of the level of chronic inflammation than a single measurement, and would be feasible to perform in clinical practice.

We assessed the proportional hazards assumption by plotting scaled Schoenfeld residuals against time. We found evidence of non-proportional hazards for many of the endpoints, with a stronger association in the first few months, so we split the follow-up time at 6 months in order to demonstrate changes in the strength of association over time.

Multiple imputation

We used two methods of multiple imputation for imputing missing data: Random Forest and normal-based MICE. For the primary analysis we used Random Forest multiple imputation, as implemented in the CALIBERrfimpute package (https://cran.r-project.org/web/packages/CALIBERrfimpute/index.html), as it can account for interactions between predictor variables without requiring them to be explicitly specified in the imputation models (6). Categorical variables were imputed using the 'rfcat' function, in which each imputed value is the prediction from a randomly chosen tree. Continuous variables were imputed using the 'rfcont' function, in which imputed values are randomly drawn from normal distributions centred on imputed means estimated using Random Forest.

The Random Forest algorithm has theoretical advantages but it is new, so we also applied the established method of normal-based MICE imputation, and verified that the results were similar. We included the following variables in imputation models:

- Complete blood count parameters: total leukocyte count, neutrophil count, lymphocyte count, eosinophil count, basophil count, monocyte count, hemoglobin concentration, platelet count. For normal-based imputation, we included leukocyte subtype counts as categorical variables (quintiles) as they would be used in the analysis, in order to avoid imposing linearity in the imputation models.
- Cardiovascular risk factors including those in the substantive models: age, age squared, sex, body mass index, blood pressure, diabetes, smoking, total cholesterol, HDL cholesterol, triglycerides, eGFR, index of multiple deprivation
- Event indicator and type of event

- Time as the marginal Nelson-Aalen cumulative hazard
- Use of statins or blood pressure lowering medication in the year before study entry
- Conditions potentially affecting blood counts: hemoglobinopathy, prior diagnosis of myelodysplasia, anemia diagnosis within 30 days prior, anemia diagnosis in following 30 days, prior diagnosis of cancer, cancer diagnosis in following 2 years, chemotherapy within 6 months prior, chemotherapy in following 3 months, renal dialysis prior, HIV diagnosis prior, steroid prescription within 3 months prior, methotrexate prescription within 3 months prior, prescription for another drug affecting the immune system within 30 days prior, infection diagnosis within 30 days prior, infection diagnosis within 30 days after, infective symptoms within 30 days prior, infective symptoms within 30 days after, splenectomy prior, immunization within 1-7 days prior
- Prior record of other comorbidities: atrial fibrillation, chronic obstructive pulmonary disease, inflammatory bowel disease, atopy, asthma, cancer, systemic autoimmune conditions

For imputing continuous measurements using Random Forest, we additionally used the last measurement before the 1-year time window before study entry, and the first measurement after study entry, along with the timing of these measurements relative to the study start date, as auxiliary variables for the imputation of that variable. Completeness of recording within 1 year prior to index date was 31.6% for HDL and total cholesterol, 65.4% for blood pressure, 47.0% for eGFR, and 31.3% for body mass index. Completeness of recording at any time (as used for imputation) was 58.9% for HDL and total cholesterol, 98.8% for blood pressure, 57.1% for eGFR and 90.6% for body mass index.

We log transformed skewed variables (HDL cholesterol, eGFR, total cholesterol and triglycerides) before imputation because both rfcont and normal-based multiple imputation functions assume a normal distribution of residuals. We exponentiated the imputed values and truncated the upper end of the distribution to the maximum value in the original data (this affected only a small number of observations, but the values were very large and would affect any normal-based linear modelling). We split the dataset was split by gender and geographical region for multiple imputation, with general practice included as a categorical variable. We generated imputations in parallel on the CALIBER high performance computing cluster. We generated 20 imputed datasets, each drawn from 20 iterations of MICE. We reviewed plots of chain means and variances of imputed variables to verify that 20 was a sufficient number of iterations. We combined the results of Cox models using Rubin's rules. The ratio of between-imputation variance to total variance ('fraction of missing information') (7) for each parameter of interest was less than 20%. We chose to generate 20 imputed datasets as a pragmatic compromise, aiming to minimise simulation error without excessive run time.

References

1. Shah AD, Denaxas S, Nicholas O, et al. Low eosinophil and low lymphocyte counts and the incidence of 12 cardiovascular diseases: a CALIBER cohort study. Open Heart. 2016;3(2):e000477. doi: 10.1136/openhrt-2016-000477.
2. Denaxas SC, George J, Herrett E, et al. Data Resource Profile: Cardiovascular disease research using linked bespoke studies and electronic health records (CALIBER). Int J Epidemiol. 2012;41(6):1625–1638. doi: 10.1093/ije/dys188.
3. Herrett E, Gallagher AM, Bhaskaran K, et al. Data Resource Profile: Clinical Practice Research Datalink (CPRD). Int J Epidemiol. 2015;44(3):827–836. doi: 10.1093/ije/dyv098.
4. Herrett E, Smeeth L, Walker L, et al. The Myocardial Ischemia National Audit Project (MINAP). Heart. 2010;96(16):1264–1267. doi: 10.1136/hrt.2009.192328.
5. Herrett E, Shah AD, Boggon R, et al. Completeness and diagnostic validity of recording acute myocardial infarction events in primary care, hospital care, disease registry, and national mortality records: cohort study. BMJ. 2013;346:f2350. doi: 10.1136/bmj.f2350.
6. Shah AD, Bartlett JW, Carpenter JR, et al. Comparison of Random Forest and parametric imputation models when imputing missing data using MICE: a CALIBER study. Am J Epidemiol. 2014;179(6):764–774. doi: 10.1093/aje/kwt312.
7. van Buuren S, Groothuis-Oudshoorn K. mice: Multivariate Imputation by Chained Equations in R. J Stat Softw. 2011;45(3):1–67.

8. Adamsson Eryd S, Smith JG, Melander O, et al. Incidence of coronary events and case fatality rate in relation to blood lymphocyte and neutrophil counts. Arterioscler Thromb Vasc Biol. 2012;32(2):533–9. doi: 10.1161/atvbaha.111.240416.
9. Zia E, Melander O, Bjorkbacka H, et al. Total and differential leucocyte counts in relation to incidence of stroke subtypes and mortality: A prospective cohort study. J Intern Med. 2012;272(3):298–304. doi: 10.1111/j.1365-2796.2012.02526.x.
10. Pfister R, Sharp SJ, Luben R, et al. Differential white blood cell count and incident heart failure in men and women in the EPIC-Norfolk study. Eur Heart J. 2012;33(4):523–30. http://dx.doi.org/10.1093/eurheartj/ehr457.
11. Bekwelem W, Lutsey PL, Loehr LR, et al. White Blood Cell Count, C-Reactive Protein, and Incident Heart Failure in the Atherosclerosis Risk in Communities (ARIC) Study. Ann Epidemiol. 2011;21(10):739–748. doi: 10.1016/j.annepidem.2011.06.005.
12. Wheeler JG, Mussolino ME, Gillum RF, et al. Associations between differential leucocyte count and incident coronary heart disease: 1764 incident cases from seven prospective studies of 30,374 individuals. Eur Heart J. 2004;25(15):1287–1292. doi: 10.1016/j.ehj.2004.05.002.
13. Gillum RF, Mussolino ME, Madans JH. Counts of neutrophils, lymphocytes, and monocytes, cause-specific mortality and coronary heart disease: The NHANES-I epidemiologic follow-up study. Ann Epidemiol. 2005;15(4):266–271.
14. Olivares R, Ducimetière P, Claude JR. Monocyte Count: A Risk Factor for Coronary Heart Disease? Am J Epidemiol. 1993;137(1):49–53.
15. Karino S, Willcox BJ, Fong K, et al. Total and differential white blood cell counts predict eight-year incident coronary heart disease in elderly Japanese-American men: the Honolulu Heart Program. Atherosclerosis. 2015;238(2):153–158. doi: 10.1016/j.atherosclerosis.2014.12.003.
16. Sweetnam PM, Thomas HF, Yarnell JW, et al. Total and differential leukocyte counts as predictors of ischemic heart disease: the Caerphilly and Speedwell studies. Am J Epidemiol. 1997;145(5):416–21.

Online Tables

Online Table 1: Cohort studies with over 2000 participants investigating neutrophil counts and incidence of cardiovascular diseases

Author, year, reference	Cohort	N patients	Examined normal range?	Clinically measured neutrophil count?	Number and type of endpoints	N events	Adjusted measure of association
Adamsson Eryd (2012) (8)	Malmo Diet and Cancer Study	27 085	No	No	1 - coronary death or myocardial infarction	1965	HR 1.13 (95% CI 1.08, 1.18) per SD (1.33 $\times 10^9$/L) higher
Zia (2012) (9)	Malmo Diet and Cancer Study	26 927	No	No	1 - cerebral infarction	1314	HR 1.3 (95% CI 1.1, 1.5) top vs bottom quartile
					2 - intracerebral hemorrhage	201	HR 0.8 (95% CI 0.5, 1.2) top vs bottom quartile
Pfister (2012) (10)	EPIC-Norfol k	16 011	No	No	1 - heart failure	935	HR (95% CI) per 109 /L higher: men 1.16 (1.09, 1.24), women 1.05 (0.97, 1.13)
Bekwelem (2011) (11)	ARIC	14 485	No	No	1 - heart failure	1647	HR 2.19 (95% 1.83, 2.61) top vs bottom quintile
Wheeler (2004) (12)	ARIC	11 305	No	No	1 - coronary death or myocardial infarction	527	RR 2.0 for top vs bottom tertile
Gillum (2005) (13)	NHEFS	4625	No	No	1 - coronary artery disease or coronary death	914	HR 1.09 (0.93, 1.29) top vs bottom tertile
Olivares (1993) (15)	Paris Prospective Study II	3659 (men only)	No	No	1 - coronary heart disease	46	RR 1.03 (95% CI 0.88, 1.19) per 10^9/L higher
Karino (2015) (15)	Honolulu Heart Program	2879	No	No	1 - acute coronary syndrome or coronary death	279	HR 1.66 (95% CI 1.11, 2.48) top vs bottom quartile (P for trend 0.01)
Sweetnam (1997) (16)	Caerphilly	2163 (men only)	No	No	1 - coronary death or myocardial infarction	143	HR 3.54 for top vs bottom quintile (P for trend 0.003)
Shah et al. (2016) (Current study)	CALIBER	775 231	Yes	Yes	12 cardio-vascular endpoints	55 004	see results

CI = confidence interval; HR = hazard ratio; RR = relative risk.

Online Table 2: Characteristics of patients and prevalence of conditions defining 'acute' and 'stable' complete blood counts

		Complete blood count (CBC) recorded while eligible for study		
	**No CBC**	**Patients excluded**	**'Acute' CBC**	**'Stable' CBC**
N patients	1 034 863	1630	154 179	621 052
Women, n (%)	455 290 (44.0%)	780 (47.9%)	94 383 (61.2%)	367 573 (59.2%)
Age, median (IQR)	N/A	53.1 (42.7–65.0)	56.2 (43.1–68.9)	51.6 (41.1–63.4)
Most deprived quintile, n (%)	220 256 (21.4%)	328 (20.2%)	27 196 (17.7%)	105 775 (17.1%)
Duration of registration before index date (years), median (IQR)	N/A	11.3 (4.09–19.1)	11.1 (4.21–20.3)	10.9 (4.22–19.6)
Median neutrophil count (IQR), $\times 10^9$/L		4.3 (3.2–5.8)	4.1 (3.1–5.4)	3.8 (3.0–4.9)
**Ethnicity, n (%):**				
White	389 590 (87.1%)	1086 (91.3%)	96 591 (92.7%)	348 443 (92.8%)
South Asian	14 697 (3.3%)	12 (1.0%)	3015 (2.9%)	10 435 (2.8%)
Black	20 471 (4.6%)	68 (5.7%)	2301 (2.2%)	7673 (2.0%)
Other	22 511 (5.0%)	24 (2.0%)	2282 (2.2%)	9038 (2.4%)
Missing	587 594 (56.8%)	440 (27.0%)	49 990 (32.4%)	245 463 (39.5%)
**Recording within 1 year before study entry:**				
HDL and total cholesterol		448 (27.5%)	40 520 (26.3%)	204 507 (32.9%)
Blood pressure		1062 (65.2%)	97 069 (63.0%)	409 775 (66.0%)
eGFR		892 (54.7%)	79 971 (51.9%)	284 628 (45.8%)
Body mass index		540 (33.1%)	47 194 (30.6%)	195 144 (31.4%)
**Person level exclusions (eMERGE):**				
HIV before index date		156 (9.6%)	0	0
Dialysis before index date		287 (17.6%)	0	0
Splenectomy before index date		1189 (72.9%)	0	0
**Conditions at the time of CBC measurement (adapted from eMERGE):**				
In hospital on date of blood test		25 (1.5%)	3080 (2.0%)	0
Vaccination within previous 7 days		53 (3.3%)	8171 (5.3%)	0
Anemia diagnosis within 30 days before		24 (1.5%)	3757 (2.4%)	0
Infection diagnosis within 30 days before		174 (10.7%)	54 251 (35.2%)	0
Infective symptoms within 30 days before		118 (7.2%)	43 178 (28.0%)	0
Prior diagnosis of myelodysplastic syndrome		7 (0.4%)	306 (0.2%)	0
Prior diagnosis of hemoglobinopathy		11 (0.7%)	2649 (1.7%)	0
Chemotherapy or G-CSF within 6 months before		19 (1.2%)	2056 (1.3%)	0
Methotrexate within 3 months before		2 (0.1%)	3077 (2.0%)	0
Steroid within 3 months before		152 (9.3%)	21 176 (13.7%)	0
Other immune drug within 3 months before		161 (9.9%)	8393 (5.4%)	0

Online Table 3: Prevalence of acute conditions by category of neutrophil count

	Below normal range	Within normal range			Above normal range
**Neutrophil count, $\times 10^9$/L**	**<2**	**2–3**	**3–6**	**6–7**	**≥7**
In hospital on date of blood test	149 (0.6%)	263 (0.2%)	1292 (0.3%)	333 (0.7%)	1043 (1.9%)
Vaccination within previous 7 days	285 (1.1%)	1575 (1.0%)	5410 (1.1%)	436 (0.9%)	465 (0.8%)
Anemia diagnosis within 30 days before	243 (0.9%)	752 (0.5%)	2103 (0.4%)	266 (0.5%)	393 (0.7%)
Infection diagnosis within 30 days before	1846 (6.9%)	9446 (6.1%)	32 356 (6.6%)	4190 (8.6%)	6413 (11.5%)
Infective symptoms within 30 days before	1470 (5.5%)	7242 (4.7%)	26 015 (5.3%)	3464 (7.1%)	4987 (8.9%)
Prior diagnosis of myelodysplastic syndrome	74 (0.3%)	80 (0.1%)	123 (0.0%)	13 (0.0%)	16 (0.0%)
Prior diagnosis of hemoglobinopathy	268 (1.0%)	668 (0.4%)	1432 (0.3%)	118 (0.2%)	163 (0.3%)
Chemotherapy or G-CSF within 6 months prior	364 (1.4%)	391 (0.3%)	865 (0.2%)	119 (0.2%)	317 (0.6%)
Methotrexate within 3 months prior	39 (0.1%)	346 (0.2%)	1935 (0.4%)	344 (0.7%)	413 (0.7%)
Steroid within 3 months prior	383 (1.4%)	2108 (1.4%)	11 601 (2.4%)	2344 (4.8%)	4740 (8.5%)
Other immune drug within 3 months prior	376 (1.4%)	1611 (1.0%)	4919 (1.0%)	635 (1.3%)	852 (1.5%)
Prior diagnosis of cancer	2089 (7.9%)	9343 (6.0%)	29 468 (6.0%)	2863 (5.9%)	3758 (6.7%)
Any acute condition	5670 (21.3%)	26 932 (17.4%)	92 969 (19.0%)	11 600 (23.7%)	17 008 (30.5%)

Online Table 4: Endpoints by category of neutrophil count

	Below normal range	Within normal range			Above normal range
**Neutrophil count, $\times 10^9$/L**	**<2**	**2–3**	**3–6**	**6–7**	**≥7**
**Initial presentation of cardiovascular disease**					
Stable angina	229 (20.7%)	1763 (22.0%)	6444 (17.5%)	570 (13.1%)	542 (11.3%)
Unstable angina	70 (6.3%)	526 (6.6%)	1918 (5.2%)	179 (4.1%)	187 (3.9%)
Coronary disease not further specified	135 (12.2%)	943 (11.8%)	3295 (9.0%)	257 (5.9%)	293 (6.1%)
Non-fatal myocardial infarction	100 (9.0%)	874 (10.9%)	4417 (12.0%)	562 (12.9%)	613 (12.8%)
Unheralded coronary death	38 (3.4%)	338 (4.2%)	2040 (5.5%)	282 (6.5%)	392 (8.2%)
Heart failure	105 (9.5%)	646 (8.1%)	4141 (11.3%)	634 (14.6%)	698 (14.6%)
Ventricular arrhythmia or sudden cardiac death	20 (1.8%)	109 (1.4%)	481 (1.3%)	60 (1.4%)	77 (1.6%)
Transient ischemic attack	109 (9.9%)	794 (9.9%)	3416 (9.3%)	380 (8.7%)	377 (7.9%)
Ischemic stroke	80 (7.2%)	532 (6.7%)	2483 (6.8%)	297 (6.8%)	298 (6.2%)
Stroke not further specified	87 (7.9%)	519 (6.5%)	2802 (7.6%)	376 (8.6%)	407 (8.5%)
Subarachnoid hemorrhage	22 (2.0%)	107 (1.3%)	338 (0.9%)	48 (1.1%)	62 (1.3%)
Intracerebral hemorrhage	29 (2.6%)	176 (2.2%)	696 (1.9%)	76 (1.7%)	83 (1.7%)
Peripheral arterial disease	66 (6.0%)	528 (6.6%)	3344 (9.1%)	508 (11.7%)	620 (13.0%)
Abdominal aortic aneurysm	16 (1.4%)	143 (1.8%)	945 (2.6%)	126 (2.9%)	136 (2.8%)
Total	1106 (100%)	7998 (100%)	36 760 (100%)	4355 (100%)	4785 (100%)
**Other deaths**					
Cancers	675 (63.4%)	2107 (55.2%)	10 025 (52.1%)	1726 (55.0%)	3060 (57.4%)
Dementia	22 (2.1%)	157 (4.1%)	1102 (5.7%)	137 (4.4%)	187 (3.5%)
Pneumonia	41 (3.8%)	201 (5.3%)	1275 (6.6%)	216 (6.9%)	331 (6.2%)
Chronic obstructive pulmonary disease	11 (1.0%)	78 (2.0%)	736 (3.8%)	178 (5.7%)	323 (6.1%)
Liver disease	65 (6.1%)	192 (5.0%)	444 (2.3%)	54 (1.7%)	114 (2.1%)
Other causes of death	251 (23.6%)	1084 (28.4%)	5651 (29.4%)	828 (26.4%)	1320 (24.7%)
Total	1065 (100%)	3819 (100%)	19 233 (100%)	3139 (100%)	5335 (100%)

Online Table 5: Cumulative incidence of initial cardiovascular presentations

Neutrophil count, ×10⁹/L	Year	Below normal range	Within normal range			Above normal range
		<2	2–3	3–6	6–7	≥7
Number at risk	0	26 588	154 863	489 143	48 849	55 788
	2	18 237	110 824	350 086	33 741	37 049
	4	11 831	74 015	234 246	22 182	24 185
	8	2470	15 391	48 195	4489	4881
Stable angina	2	0.48 (0.39–0.57)	0.65 (0.61–0.69)	0.77 (0.74–0.79)	0.73 (0.65–0.81)	0.50 (0.44–0.57)
	4	0.87 (0.74–1.00)	1.12 (1.06–1.18)	1.27 (1.23–1.30)	1.16 (1.06–1.27)	0.88 (0.79–0.97)
	8	1.52 (1.29–1.76)	1.89 (1.79–2.00)	2.12 (2.06–2.18)	1.81 (1.64–1.99)	1.68 (1.51–1.85)
Unstable angina or CHD NOS	2	0.34 (0.27–0.42)	0.44 (0.40–0.47)	0.49 (0.47–0.51)	0.46 (0.40–0.52)	0.44 (0.38–0.50)
	4	0.68 (0.56–0.80)	0.81 (0.76–0.86)	0.91 (0.88–0.94)	0.80 (0.71–0.89)	0.80 (0.71–0.88)
	8	1.47 (1.21–1.72)	1.80 (1.68–1.91)	1.91 (1.85–1.97)	1.46 (1.30–1.63)	1.51 (1.34–1.67)
Non-fatal myocardial infarction	2	0.14 (0.10–0.19)	0.24 (0.21–0.26)	0.39 (0.37–0.41)	0.50 (0.44–0.57)	0.55 (0.49–0.62)
	4	0.30 (0.22–0.38)	0.49 (0.45–0.53)	0.78 (0.76–0.81)	1.00 (0.90–1.11)	1.03 (0.93–1.12)
	8	0.87 (0.65–1.09)	1.07 (0.99–1.16)	1.61 (1.56–1.67)	2.17 (1.95–2.38)	1.87 (1.70–2.05)
Unheralded coronary death	2	0.08 (0.04–0.11)	0.09 (0.07–0.10)	0.18 (0.17–0.20)	0.28 (0.23–0.33)	0.41 (0.35–0.46)
	4	0.13 (0.08–0.18)	0.19 (0.17–0.22)	0.37 (0.35–0.39)	0.52 (0.45–0.59)	0.67 (0.59–0.75)
	8	0.26 (0.16–0.36)	0.42 (0.37–0.48)	0.76 (0.72–0.80)	1.06 (0.91–1.21)	1.14 (1.01–1.28)
Heart failure	2	0.23 (0.17–0.29)	0.20 (0.18–0.23)	0.46 (0.44–0.48)	0.86 (0.77–0.95)	0.84 (0.76–0.92)
	4	0.32 (0.25–0.40)	0.36 (0.32–0.39)	0.77 (0.74–0.80)	1.25 (1.14–1.36)	1.23 (1.12–1.33)
	8	0.69 (0.52–0.85)	0.77 (0.69–0.84)	1.43 (1.38–1.48)	2.04 (1.85–2.23)	1.83 (1.67–1.99)
Ventricular arrhythmia or SCD	2	0.03 (0.01–0.05)	0.04 (0.03–0.05)	0.04 (0.04–0.05)	0.07 (0.05–0.10)	0.08 (0.05–0.10)
	4	0.05 (0.02–0.08)	0.07 (0.05–0.08)	0.09 (0.08–0.10)	0.10 (0.07–0.13)	0.12 (0.09–0.15)
	8	0.18 (0.09–0.28)	0.12 (0.09–0.15)	0.18 (0.16–0.20)	0.21 (0.15–0.28)	0.24 (0.17–0.31)
Transient ischemic attack	2	0.23 (0.17–0.29)	0.24 (0.21–0.27)	0.34 (0.32–0.36)	0.41 (0.35–0.47)	0.40 (0.34–0.45)
	4	0.41 (0.32–0.50)	0.45 (0.42–0.49)	0.62 (0.60–0.65)	0.72 (0.63–0.81)	0.69 (0.61–0.77)
	8	0.73 (0.56–0.90)	0.93 (0.85–1.00)	1.23 (1.18–1.28)	1.29 (1.13–1.44)	1.12 (0.99–1.26)
Ischemic or unspecified stroke	2	0.27 (0.21–0.34)	0.28 (0.26–0.31)	0.50 (0.48–0.52)	0.74 (0.66–0.82)	0.74 (0.66–0.81)
	4	0.52 (0.42–0.63)	0.57 (0.53–0.62)	0.96 (0.93–1.00)	1.24 (1.12–1.35)	1.16 (1.06–1.26)
	8	1.21 (0.98–1.44)	1.35 (1.25–1.45)	1.94 (1.87–2.00)	2.38 (2.16–2.60)	2.05 (1.87–2.24)
Subarachnoid hemorrhage	2	0.04 (0.02–0.07)	0.03 (0.02–0.04)	0.03 (0.02–0.03)	0.06 (0.03–0.08)	0.06 (0.04–0.08)
	4	0.10 (0.05–0.14)	0.07 (0.05–0.09)	0.06 (0.05–0.07)	0.09 (0.06–0.12)	0.10 (0.07–0.13)
	8	0.17 (0.08–0.26)	0.12 (0.09–0.14)	0.13 (0.11–0.14)	0.16 (0.11–0.22)	0.19 (0.13–0.24)
Intracerebral hemorrhage	2	0.05 (0.02–0.07)	0.04 (0.03–0.05)	0.07 (0.06–0.07)	0.07 (0.04–0.09)	0.07 (0.05–0.09)
	4	0.13 (0.07–0.18)	0.10 (0.08–0.12)	0.12 (0.11–0.13)	0.14 (0.10–0.18)	0.14 (0.10–0.17)
	8	0.19 (0.11–0.28)	0.22 (0.18–0.26)	0.26 (0.24–0.29)	0.30 (0.21–0.39)	0.26 (0.19–0.33)
Peripheral arterial disease	2	0.11 (0.07–0.15)	0.15 (0.13–0.17)	0.35 (0.33–0.37)	0.58 (0.51–0.65)	0.61 (0.55–0.68)
	4	0.20 (0.14–0.27)	0.31 (0.28–0.34)	0.63 (0.60–0.65)	0.98 (0.88–1.09)	1.07 (0.97–1.17)
	8	0.51 (0.36–0.67)	0.62 (0.55–0.68)	1.18 (1.13–1.23)	1.75 (1.57–1.93)	1.82 (1.65–1.99)
Abdominal aortic aneurysm	2	0.02 (0.00–0.04)	0.04 (0.03–0.05)	0.09 (0.08–0.10)	0.17 (0.13–0.21)	0.16 (0.13–0.20)
	4	0.07 (0.03–0.11)	0.08 (0.06–0.09)	0.18 (0.16–0.19)	0.27 (0.22–0.32)	0.24 (0.20–0.29)
	8	0.11 (0.05–0.17)	0.18 (0.15–0.22)	0.34 (0.32–0.37)	0.39 (0.31–0.47)	0.35 (0.28–0.43)

CHD NOS, coronary heart disease not otherwise specified; SCD, sudden cardiac death. Estimates (%) based on unadjusted cumulative incidence curves accounting for competing risks.

Online Figures

Online Figure 1: Patient flow diagram

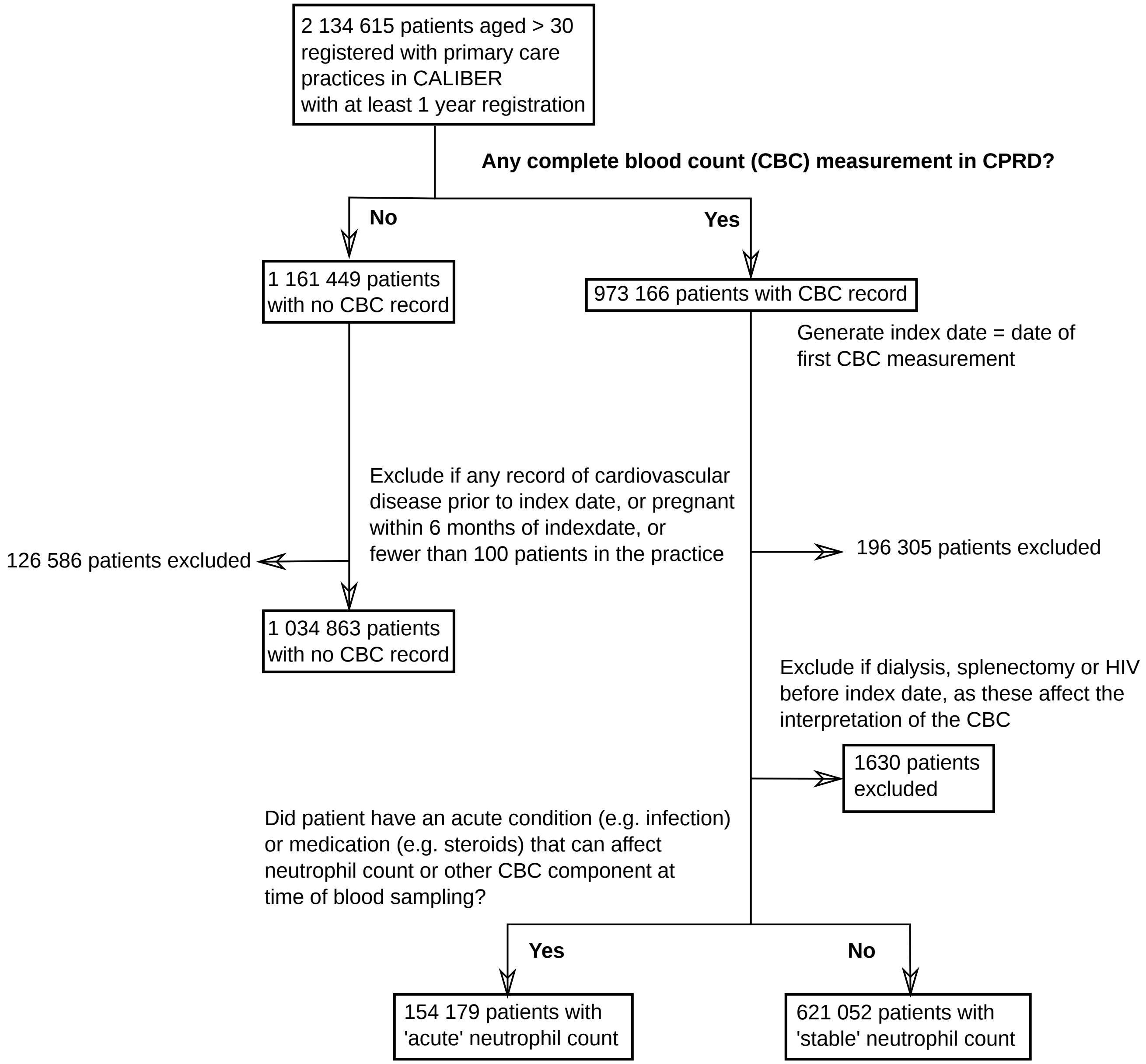

Online Figure 2: Age and sex adjusted hazard ratios for the association of neutrophil counts with different initial presentations of cardiovascular diseases

Hazard ratios adjusted for age and sex. The normal range is shaded. P values * < 0.05, ** < 0.0036, *** < 0.0001

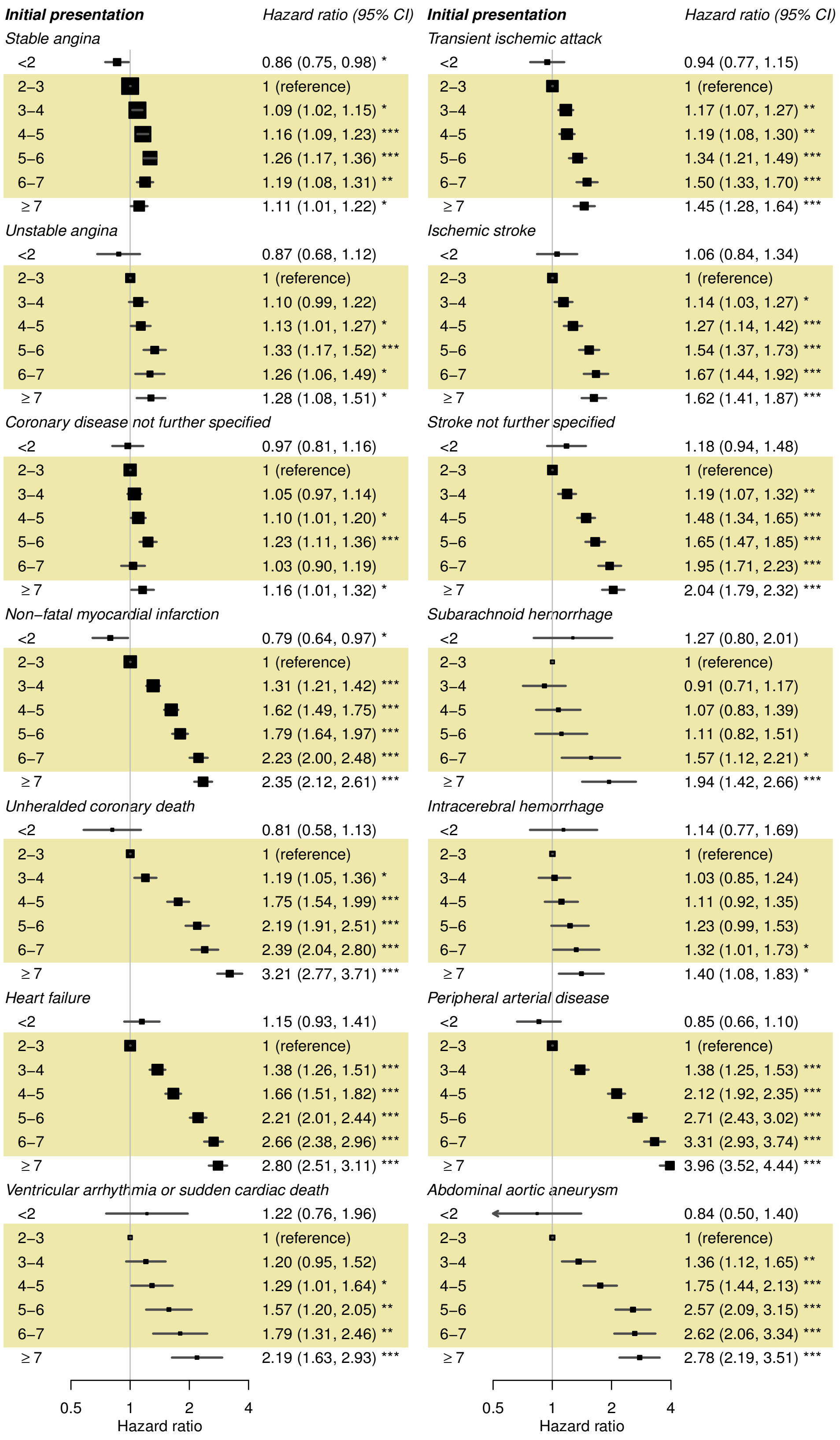

Online Figure 3: Association of neutrophil counts with composite cardiovascular disease and non-cardiovascular death

Hazard ratios are adjusted for age, sex, deprivation, ethnicity, smoking, diabetes, systolic blood pressure, blood pressure medication, body mass index, total cholesterol, HDL cholesterol, statin use, eGFR, atrial fibrillation, autoimmune conditions, inflammatory bowel disease, COPD, cancer, and acute conditions at the time of blood testing. P values * < 0.05, ** < 0.0036, *** < 0.0001

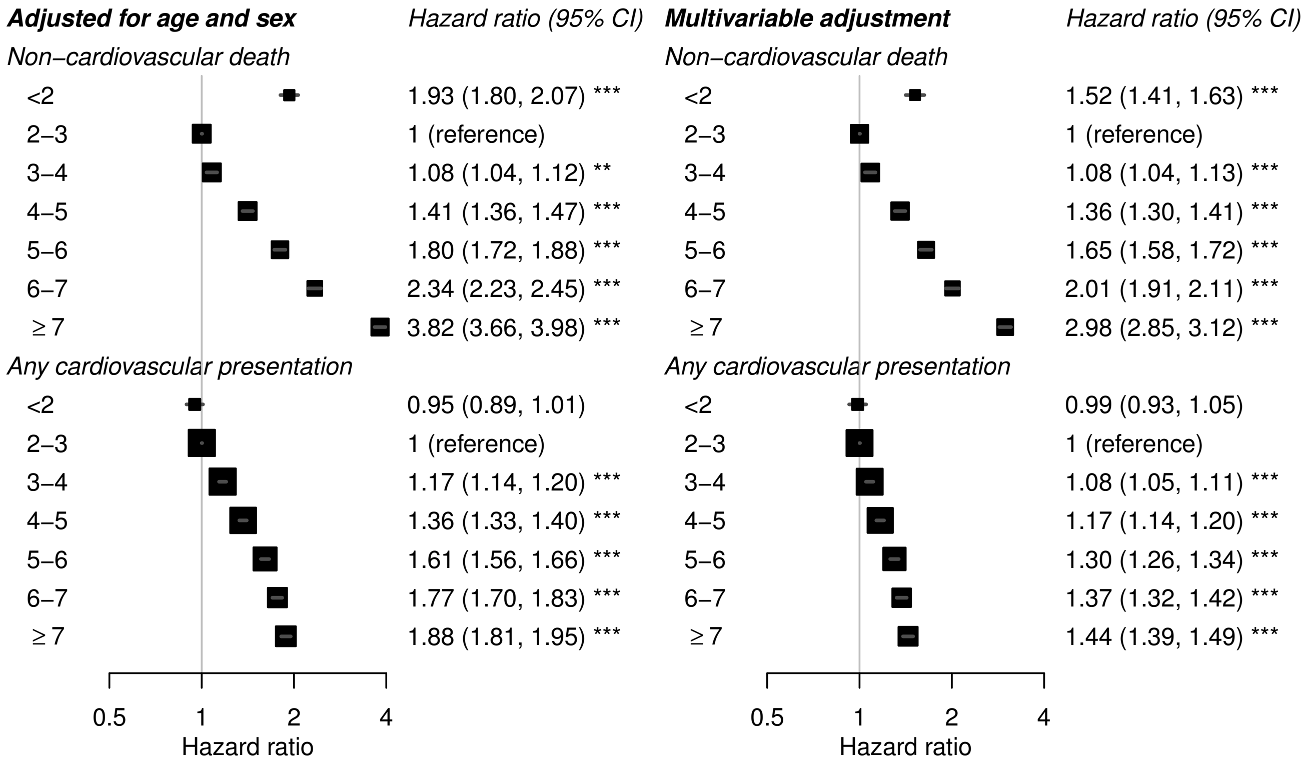

Online Figure 4: Association of neutrophil counts with all-cause mortality

Hazard ratios are adjusted for age, sex, deprivation, ethnicity, smoking, diabetes, systolic blood pressure, blood pressure medication, body mass index, total cholesterol, HDL cholesterol, statin use, eGFR, atrial fibrillation, autoimmune conditions, inflammatory bowel disease, COPD, cancer, and acute conditions at the time of blood testing. P values * < 0.05, ** < 0.0036, *** < 0.0001

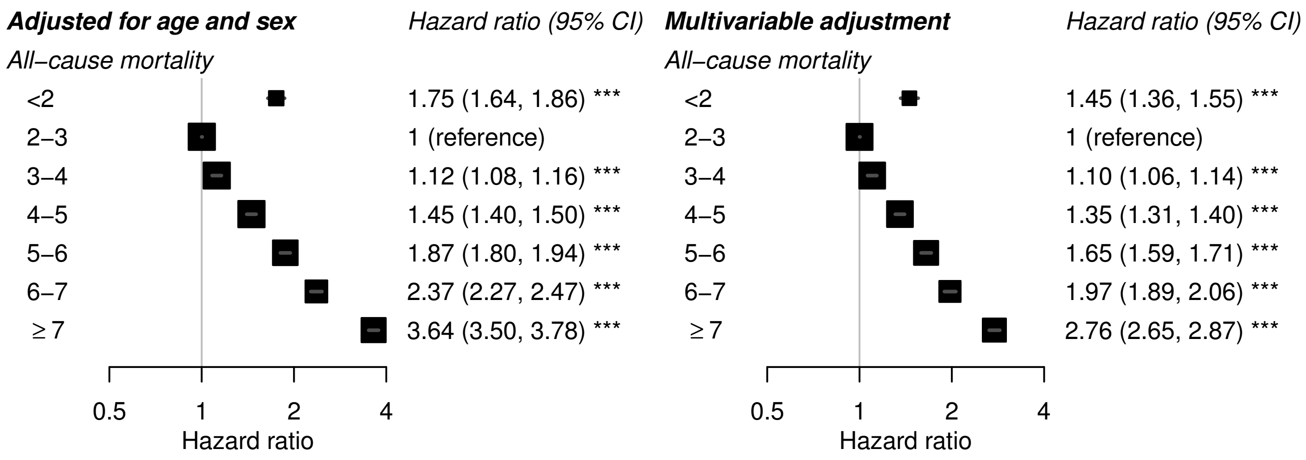

Online Figure 5: Linear association of neutrophil counts with different initial presentations of cardiovascular disease

Hazard ratios are adjusted for age, sex, deprivation, ethnicity, smoking, diabetes, systolic blood pressure, blood pressure medication, body mass index, total cholesterol, HDL cholesterol, statin use, eGFR, atrial fibrillation, autoimmune conditions, inflammatory bowel disease, COPD, cancer, and acute conditions at the time of blood testing. P values * < 0.05, ** < 0.0036, *** < 0.0001

Initial presentation	*Hazard ratio (95% CI)*	*P value for interaction*
Stable angina		
All patients	0.99 (0.98, 1.00)	0.026
Neutrophils confined to normal range	1.01 (0.99, 1.03)	
Unstable angina		
All patients	1.01 (0.98, 1.03)	0.57
Neutrophils confined to normal range	1.01 (0.98, 1.05)	
Coronary disease not further specified		
All patients	1.00 (0.98, 1.02)	0.28
Neutrophils confined to normal range	0.99 (0.97, 1.02)	
Non–fatal myocardial infarction		
All patients	1.07 (1.05, 1.08) ***	0.0018
Neutrophils confined to normal range	1.11 (1.08, 1.13) ***	
Unheralded coronary death		
All patients	1.11 (1.09, 1.12) ***	0.014
Neutrophils confined to normal range	1.20 (1.16, 1.24) ***	
Heart failure		
All patients	1.08 (1.07, 1.09) ***	<0.0001
Neutrophils confined to normal range	1.20 (1.17, 1.23) ***	
Ventricular arrhythmia or sudden cardiac death		
All patients	1.06 (1.02, 1.10) **	0.71
Neutrophils confined to normal range	1.09 (1.02, 1.17) *	
Transient ischemic attack		
All patients	1.03 (1.01, 1.05) **	0.08
Neutrophils confined to normal range	1.05 (1.02, 1.08) **	
Ischemic stroke		
All patients	1.04 (1.02, 1.06) ***	0.031
Neutrophils confined to normal range	1.09 (1.05, 1.12) ***	
Stroke not further specified		
All patients	1.07 (1.05, 1.08) ***	0.00082
Neutrophils confined to normal range	1.14 (1.11, 1.18) ***	
Subarachnoid hemorrhage		
All patients	1.04 (0.99, 1.09)	0.038
Neutrophils confined to normal range	1.02 (0.94, 1.10)	
Intracerebral hemorrhage		
All patients	1.02 (0.99, 1.06)	0.89
Neutrophils confined to normal range	1.04 (0.98, 1.10)	
Peripheral arterial disease		
All patients	1.10 (1.09, 1.12) ***	<0.0001
Neutrophils confined to normal range	1.20 (1.17, 1.23) ***	
Abdominal aortic aneurysm		
All patients	1.07 (1.04, 1.10) ***	0.00021
Neutrophils confined to normal range	1.19 (1.13, 1.25) ***	

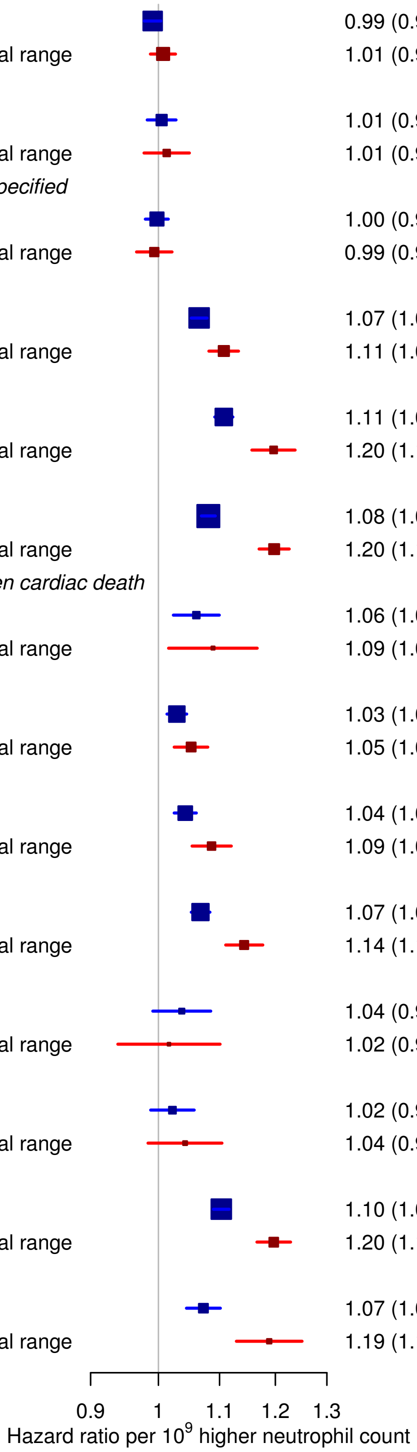

Online Figure 6: Linear association of neutrophil counts with different initial presentations of cardiovascular disease, by smoking status

Hazard ratios are adjusted for age, sex, deprivation, ethnicity, smoking, diabetes, systolic blood pressure, blood pressure medication, body mass index, total cholesterol, HDL cholesterol, statin use, eGFR, atrial fibrillation, autoimmune conditions, inflammatory bowel disease, COPD, cancer, and acute conditions at the time of blood testing. P values * < 0.05, ** < 0.0036, *** < 0.0001

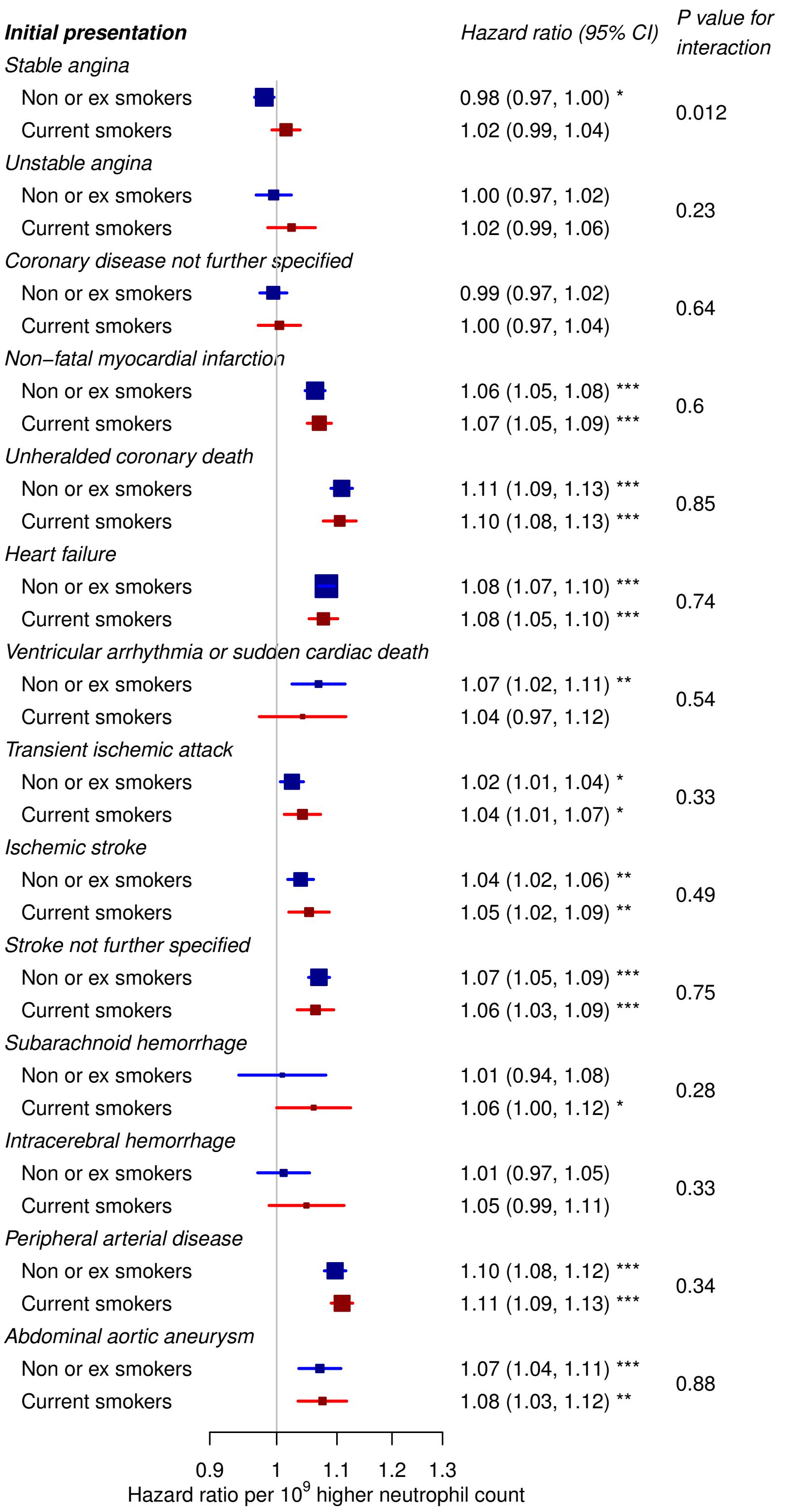

Online Figure 7: Linear association of neutrophil counts with different initial presentations of cardiovascular disease, with and without adjustment for eosinophil and lymphocyte counts

'Standard adjustment' comprises adjustment for age, sex, deprivation, ethnicity, smoking, diabetes, systolic blood pressure, blood pressure medication, body mass index, total cholesterol, HDL cholesterol, statin use, eGFR, atrial fibrillation, autoimmune conditions, inflammatory bowel disease, COPD, cancer, and acute conditions at the time of blood testing. P values * < 0.05, ** < 0.0036, *** < 0.0001

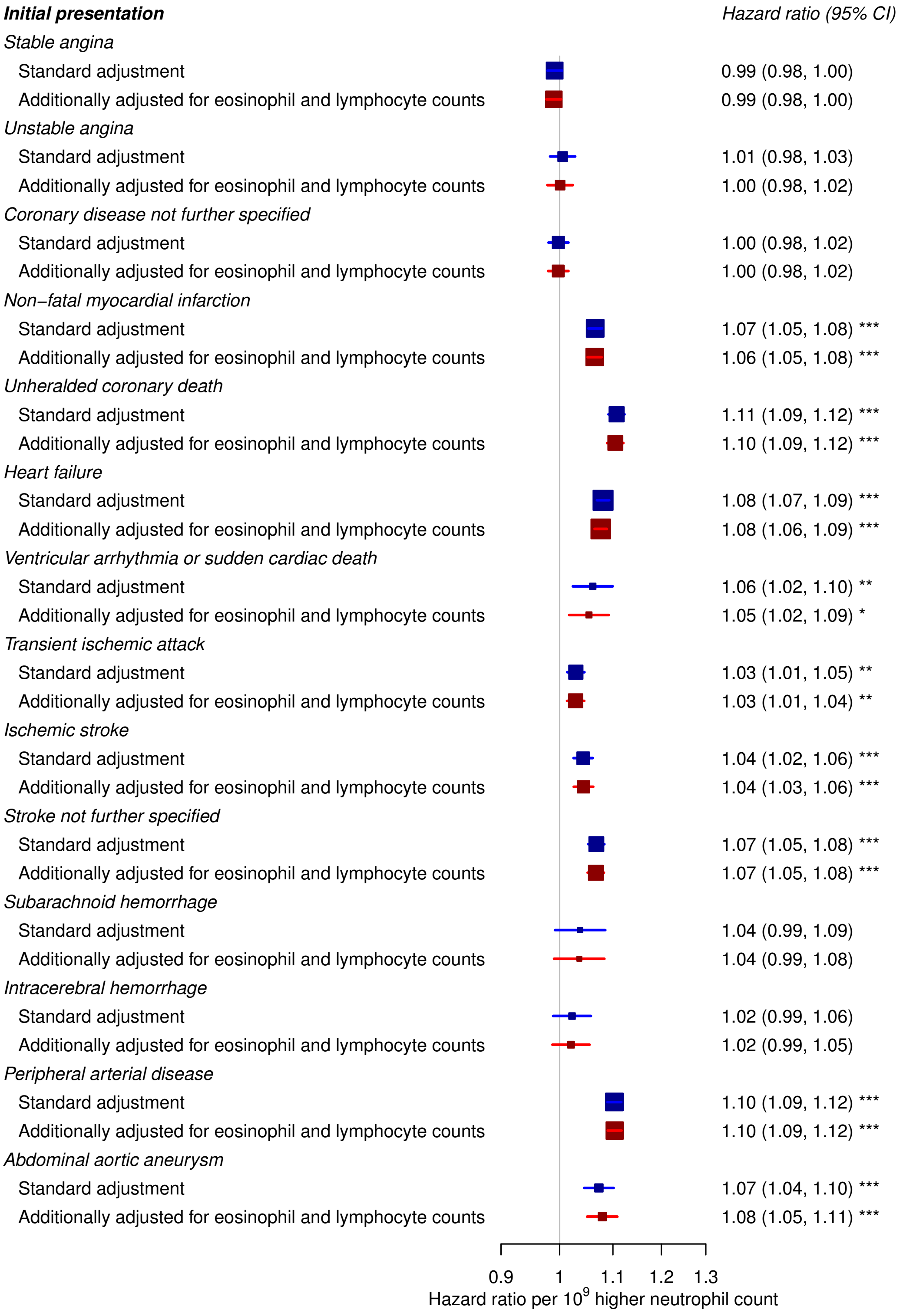

Online Figure 8: Binned scatterplot showing difference between two consecutive neutrophil counts taken when a patient was clinically 'stable'

N = 395 133. Correlation coefficient = 0.568. Standard deviation of differences = 1.67×10^9/L. The solid line is lowess smoothed mean and the dotted lines are lowess smoothed 2.5% and 97.5% centiles. Median time between blood tests is 1.37 years.

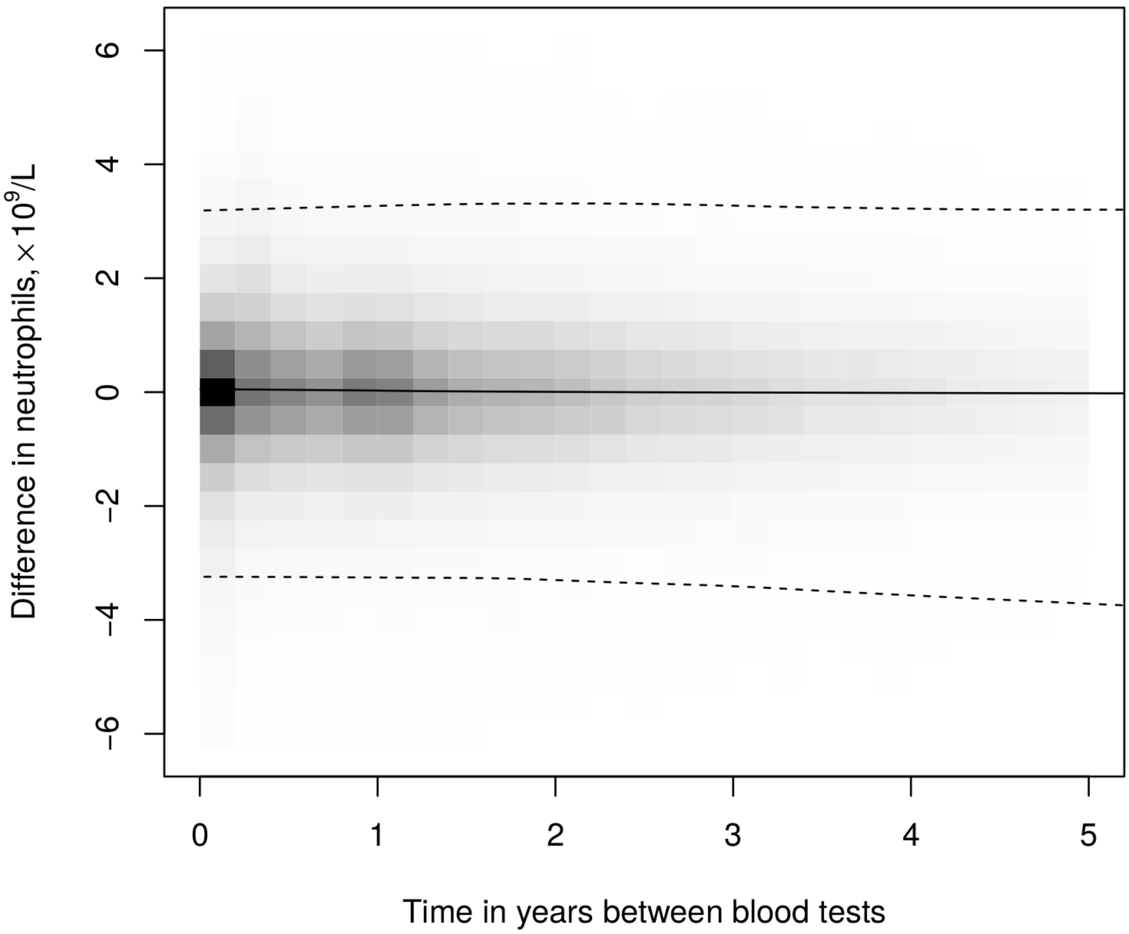

Online Figure 9: Linear association of neutrophil counts with different initial presentations of cardiovascular disease, by time since measurement

Hazard ratios are adjusted for age, sex, deprivation, ethnicity, smoking, diabetes, systolic blood pressure, blood pressure medication, body mass index, total cholesterol, HDL cholesterol, statin use, eGFR, atrial fibrillation, autoimmune conditions, inflammatory bowel disease, COPD, cancer, and acute conditions at the time of blood testing. P values * < 0.05, ** < 0.0036, *** < 0.0001

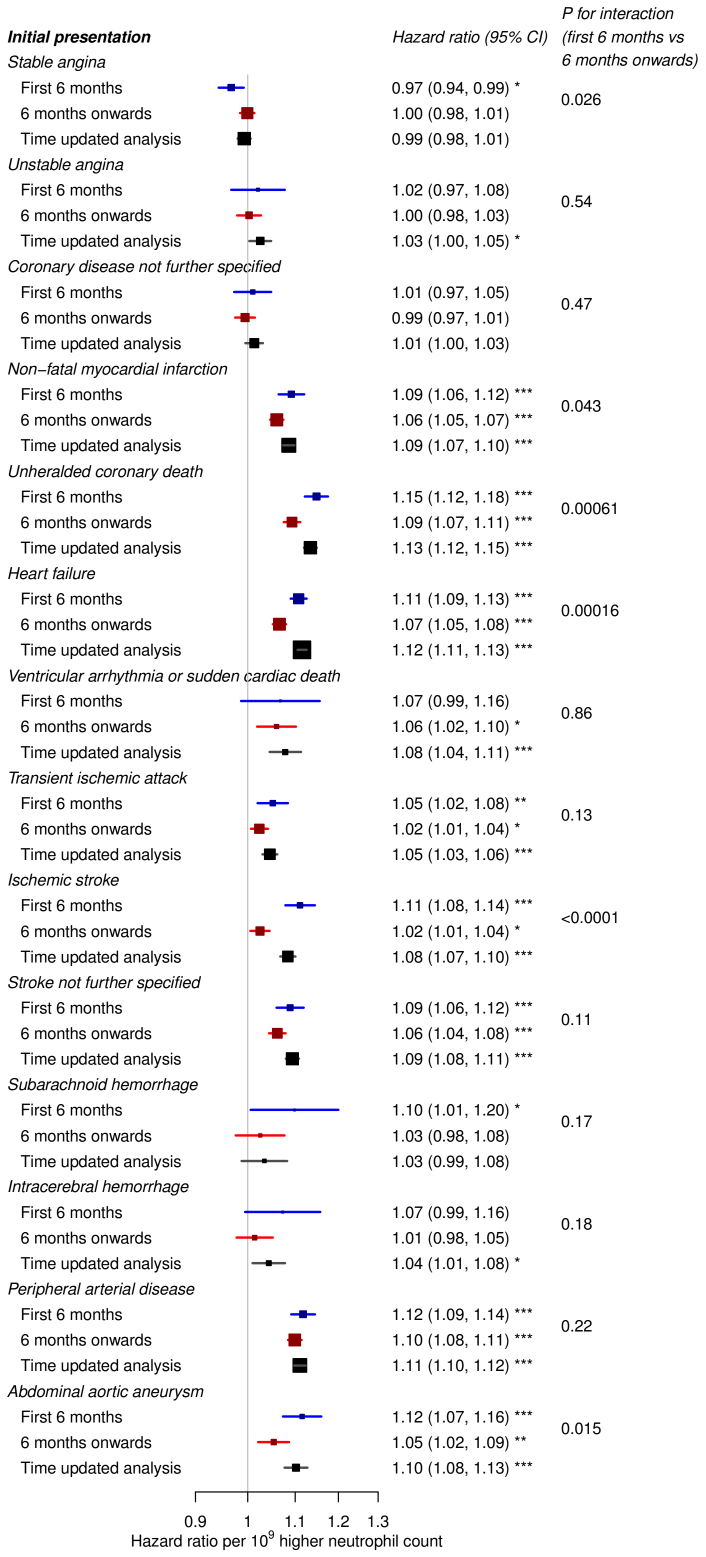

Online Figure 10: Linear association of neutrophil counts with different initial presentations of cardiovascular disease, by age group

Hazard ratios are adjusted for age, sex, deprivation, ethnicity, smoking, diabetes, systolic blood pressure, blood pressure medication, body mass index, total cholesterol, HDL cholesterol, statin use, eGFR, atrial fibrillation, autoimmune conditions, inflammatory bowel disease, COPD, cancer, and acute conditions at the time of blood testing. P values * < 0.05, ** < 0.0036, *** < 0.0001

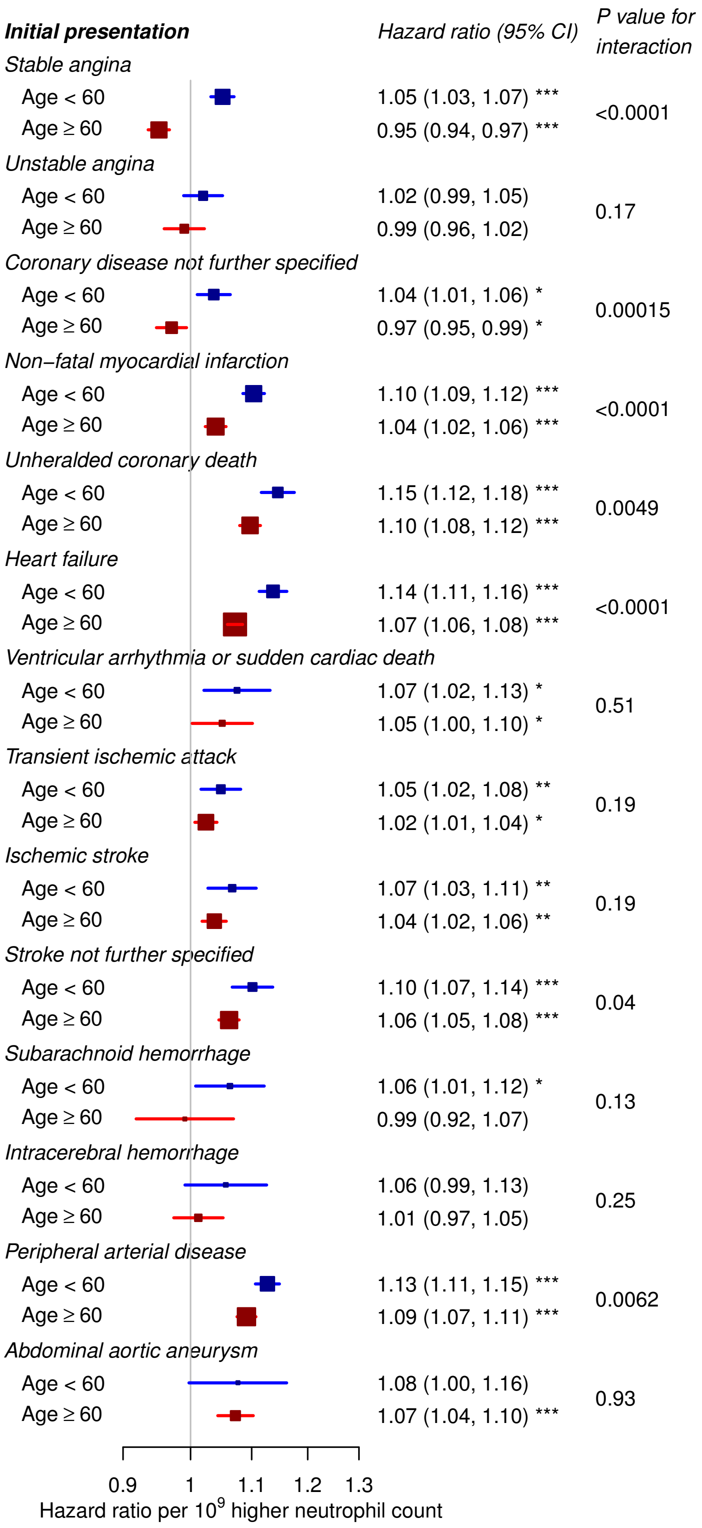

Online Figure 11: Linear association of neutrophil counts with different initial presentations of cardiovascular disease, by sex

Hazard ratios are adjusted for age, sex, deprivation, ethnicity, smoking, diabetes, systolic blood pressure, blood pressure medication, body mass index, total cholesterol, HDL cholesterol, statin use, eGFR, atrial fibrillation, autoimmune conditions, inflammatory bowel disease, COPD, cancer, and acute conditions at the time of blood testing. P values * < 0.05, ** < 0.0036, *** < 0.0001

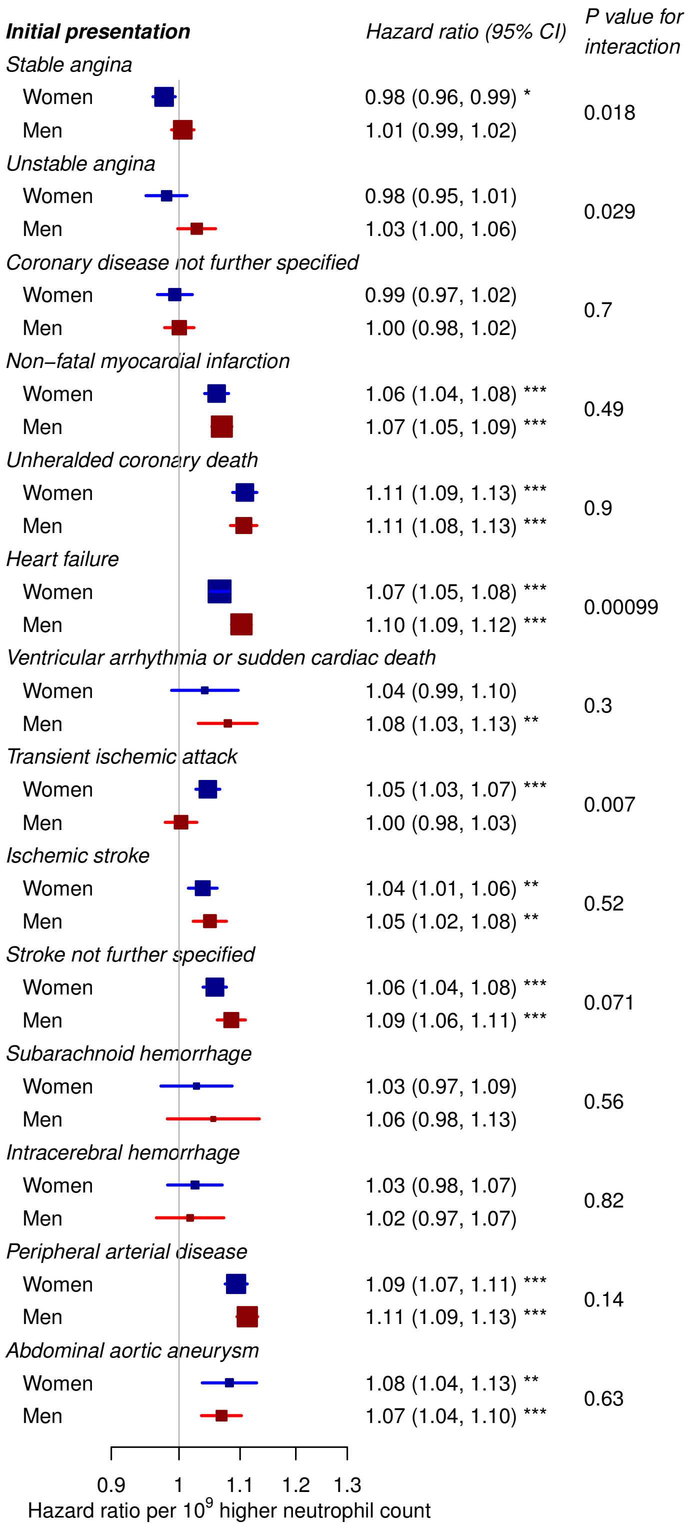

Online Figure 12: Association of quintiles of monocyte counts with different initial presentations of cardiovascular disease

Hazard ratios are adjusted for age, sex, deprivation, ethnicity, smoking, diabetes, systolic blood pressure, blood pressure medication, body mass index, total cholesterol, HDL cholesterol, statin use, eGFR, atrial fibrillation, autoimmune conditions, inflammatory bowel disease, COPD, cancer, and acute conditions at the time of blood testing. P values * < 0.05, ** < 0.0036, *** < 0.0001

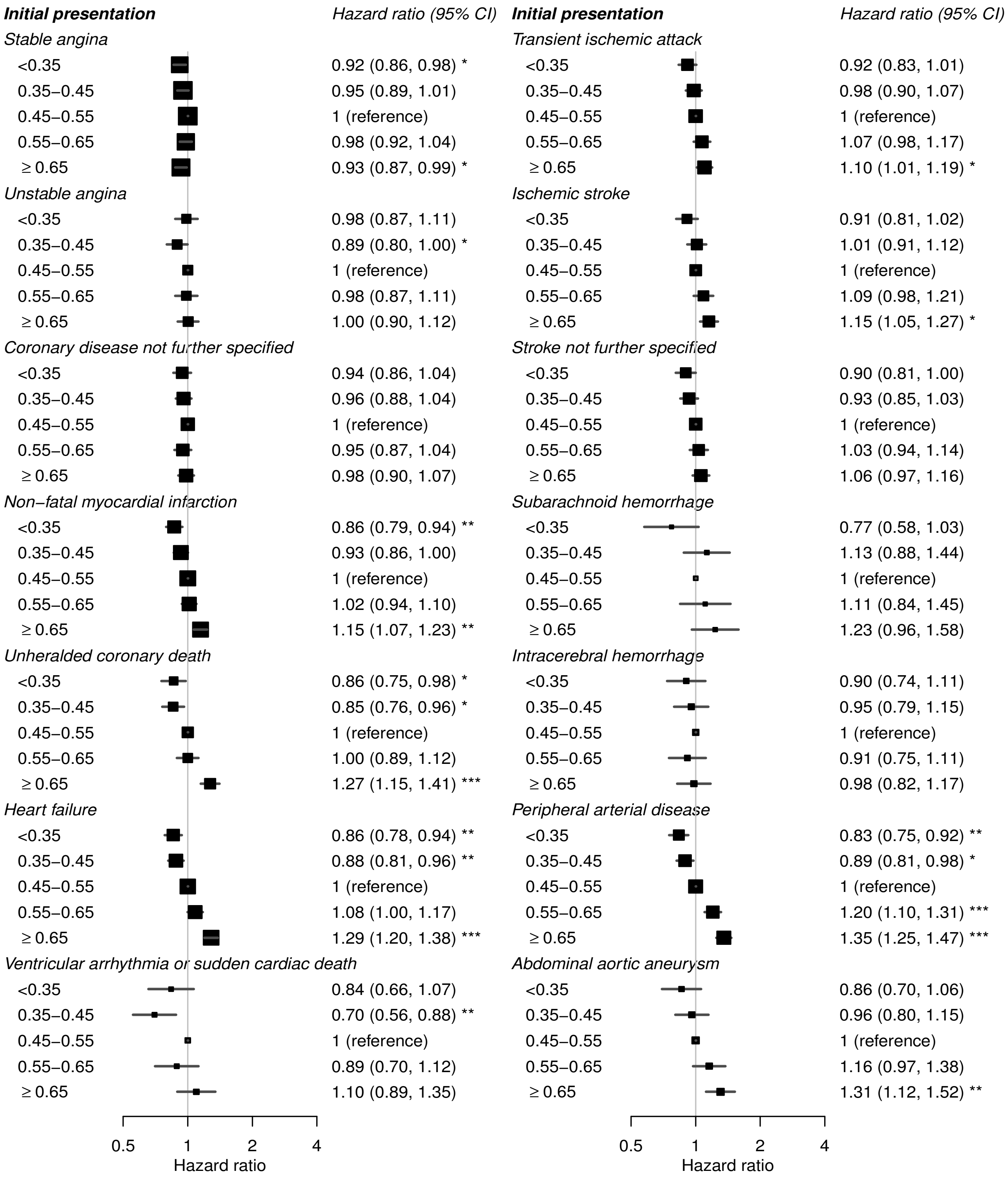

Supplement: Online Data [file mmc2.pdf]
